# Abstract

**DOI:** 10.1111/anec.70099

**Published:** 2025-07-07

## 4579

### Deep Learning ECG Image Platform for Cardiac Diagnosis and Risk Stratification

#### Boroumand Zeidaabadi ^1^, Konstantinos Patlatzoglou^1^, Libor Pastika^1^, Joseph Barker^1^, Nicholas S. Peters^1^, Daniel B. Kramer^2^, Jonathan W. Waks^3^, Fu Siong Ng^1^, Arunashis Sau^1^

##### ^1^National Heart and Lung Institute, Imperial College London, United Kingdom, ^2^Beth Israel Deaconess Medical Center, Boston, United States, ^3^Harvard‐Thorndike Electrophysiology Institute and Arrhythmia Service, Boston, United States

**Introduction:** Early detection of cardiovascular disease and accurate risk prediction are critical for improving patient outcomes. While AI‐enhanced ECG (AI‐ECG) tools show great promise, many depend on digital signal input, limiting their clinical utility in environments lacking. This study introduces an image‐based AI‐ECG platform for cardiac screening and risk prediction.

**Methods:** We developed deep learning models using over 1.1 million ECG images from 189,538 patients in a secondary care dataset. Model performance was validated internally and externally across multiple cohorts: CODE (*n* = 645,380), SaMi‐Trop (*n* = 1,022), and UK Biobank (*n* = 42,386). Robustness was tested using photographed ECGs, lead position variation, simulated artifacts, and incomplete leads.

**Results:** The platform demonstrated strong diagnostic capabilities, including detection of reduced ejection fraction (AUC 0.896) and valvular heart diseases: aortic stenosis (AUC 0.869), tricuspid regurgitation (0.850), mitral regurgitation (0.826), and aortic regurgitation (0.775). Mortality prediction showed strong performance across all cohorts (c‐statistic 0.818 [0.814–0.821] in BIDMC, 0.832 [0.814–0.850] in photographed images, 0.764 [0.761–0.768] in CODE and 0.770 [0.725–0.816] in SaMi‐Trop). Future cardiac conditions were accurately predicted in internal validation: atrial fibrillation (0.770 [0.764–0.776]), heart failure (0.809 [0.805–0.814]), ventricular arrhythmia (0.800 [0.788–0.814]), atherosclerotic cardiovascular disease (0.758 [0.752–0.765]), and complete heart block (0.855 [0.840–0.870]). High‐risk quartiles consistently showed significantly elevated event risks. Saliency mapping revealed interpretable and condition‐specific activation patterns.

**Conclusion:** This image‐based AI‐ECG platform offers robust, scalable, and interpretable cardiac risk prediction using standard ECG images. Its generalisability across diverse cohorts and image types makes it suitable for global clinical deployment, particularly in low‐resource settings.

## 4607

### AI‐ECG for Normal ECG Detection in a Large‐Scale Telehealth Network

#### Antonio Luiz Ribeiro ^1^, Petrus Abreu^1^, Antonio Horta Ribeiro^2^, Gabriela Paixão^1^, Gomes Paulo^1^

##### ^1^Federal University of Minas Gerais—UFMG, Belo Horizonte, Brazil, ^2^Department of Information Technology, Uppsala University, Uppsala, Sweden

**Introduction:** The Telehealth Network of Minas Gerais (TNMG) provides tele‐electrocardiogram (tele‐ECG) services to Brazil's public health system (SUS), analyzing approximately 7,000 ECGs daily. Since most ECGs are normal, an automated system for identifying them could optimize cardiologist workload and improve efficiency. This study aimed to develop and evaluate an artificial intelligence (AI) model for high‐precision classification of normal ECGs.

**Methods:** We trained a deep neural network (DNN) using 2,933,600 ECGs labeled by TNMG cardiologists (81% training, 19% validation). The model used 8 leads (I, II, V1–V6), resampled to 400 Hz and standardized to 4096 samples per lead. The architecture included five residual blocks and a sigmoid‐activated output layer to produce a probability score for normal ECG classification. Retrospective evaluation was performed on 8,933 ECGs with ≥ 2 concordant cardiologist interpretations. Prospective testing used 10,369 ECGs integrated into TNMG's live workflow, with cardiologists blinded to model output. Two thresholds were tested: one optimized for ≥ 95% precision (precision‐optimized), and one for the best F1 score (F1‐optimized).

**Results:** The DNN achieved an AUC of 0.933 in retrospective and 0.918 in prospective analysis. At the precision‐optimized threshold, precision reached 0.968. The F1‐optimized threshold yielded an F1 score of 0.483, with overall metrics remaining stable in both phases.

**Conclusion:** The DNN accurately detects normal ECGs with high precision, enabling its integration into TNMG's tele‐ECG platform. This system has the potential to reduce workload, improve access to care, and support national and international scaling.

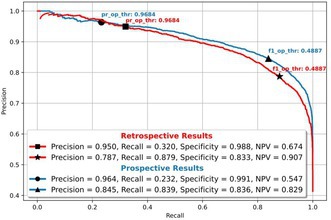

## 4565

### Pre‐Implant AI‐ECG Age as a Predictor of Survival Following Cardiac Resynchronization Therapy

#### Abhishek Deshmukh^1^

##### ^1^Mayo Clinic, Rochester, Minnesota, United States

**Introduction:** Cardiac resynchronization therapy (CRT) selection criteria are well‐defined, yet there is an ongoing need for refined predictors of post‐implant outcomes to identify responders. The advent of artificial intelligence‐enabled electrocardiography (AI‐ECG) offers a novel opportunity to evaluate biological age, potentially outperforming chronologic age in assessing physiological health. We aimed to determine whether pre‐implant AI‐ECG‐derived age, as a surrogate for biological age, can independently predict survival following CRT.

**Methods:** We conducted a retrospective review of patients receiving a de‐novo CRT‐D at Mayo Clinic (January 2001–September 2022). Inclusion criteria were left ventricular ejection fraction (LVEF) ≤ 35%, QRS duration ≥ 120 ms, and CRT‐D implantation. AI‐ECG age was extracted from the most recent pre‐implant ECG, and the delta age (ECG age minus chronologic age) was calculated. Survival analyses were performed using accelerated failure time models with a log‐logistic distribution. Time ratios (TR) were calculated to represent survival changes per unit increment of each variable. Multivariable analyses incorporated forward and backward selection of key variables including chronologic age, delta age, LVEF, QRS duration, QRS morphology, cardiomyopathy type, gender, and comorbidities such as type 2 diabetes mellitus, hypertension, and CKD stage ≥ 3.

**Results:** Four hundred sixty‐four patients were included in the analysis. The final multivariable model retained variables such as chronologic age, cardiomyopathy etiology, delta age, and the presence of type 2 diabetes mellitus, hypertension, and CKD stage ≥ 3. Multivariable analysis demonstrated a 37% survival decrease per 10‐year increase in the delta age (TR 0.963, *p* = 0.007). A lower ECG age compared to the chronological age thus portended increased survival. One hundred fifty‐five patients had follow‐up ECGs 3–6 months post implant. A multivariable analysis with stepwise selection of variables in this cohort demonstrated no correlation between change in the delta age pre‐and post‐implant with post‐implant survival.

**Conclusion:** Pre‐implant AI‐ECG derived delta age emerges as a robust, independent predictor of survival post‐CRT. Lower AI‐ECG age compared to chronologic age may reflect a lower burden of cardiac disease and enhanced physiological reserve. These findings underscore the potential of AI‐driven tools to optimize CRT patient selection and personalize therapeutic strategies.

## 4598

### Detection of Paroxysmal Atrial Fibrillation During Sinus Rhythm Using P‐Wave Loop Vectorcardiographic Indices

#### Evangelia Myrovali ^1^, Georgios Giannopoulos^1^, Dimitrios Hristu‐Varsakelis^2^, Dimitrios Tachmatzidis^1^, Vassilios Vassilikos^1^

##### ^1^3rd Cardiology Department, Aristotle University of Thessaloniki, Hippokration General Hospital, Thessaloniki 54642, Greece, ^2^Department of Applied Informatics, University of Macedonia, Thessaloniki 54006, Greece

**Background:** The P‐loop of the vectorcardiogram (VCG) reflects atrial electrical activity, where changes in vector direction and loop shape can be conceived as descriptors of the spatiotemporal characteristics of the atrial electrical cycle. The purpose of this study was to identify patients with paroxysmal atrial fibrillation (PAF) analyzing their sinus rhythm VCGs, specifically the geometry of their P‐wave loops. Detecting PAF from sinus rhythm recordings is especially relevant in particular clinical scenarios, such as cryptogenic stroke.

**Methods:** We compared patients with known PAF to healthy subjects. Cluster pair matching was used to ensure group similarity. We recorded 3 orthogonal VCG leads at 1000 Hz for 10 min during SR. We extracted P‐waves from each lead to construct per‐beat 3D P‐wave loops. For each loop, we computed the mean ± SD of three eigenvalues and a flatness index for each subject's loops. To calculate those indices, we applied singular value decomposition (SVD) to the 3D coordinates of each loop samples. SVD produces orthogonal vectors ranked by variance, with λ₁ > λ₂ > λ₃. Flatness index was calculated as 1 minus the ratio of λ3 to the sum of all eigenvalues (1 corresponding to a completely flat loop).

**Results:** We analyzed 69 patients with known PAF and 59 healthy subjects. The two groups did not differ significantly in terms of age, sex, left ventricular ejection fraction, history of coronary heart disease or heart failure and use of relevant medication (antiarrhythmics, beta‐blockers etc). We observed higher values in λ1, λ2, and λ3 in healthy subjects as compared to PAF patients (see Table for specific values and *p* values for between‐groups comparisons), as well as a significantly lower flatness index (*p* = 0.022).

**Conclusion:** Our results suggest that the electrical propagation pattern in the atria differs in individuals with PAF who are in sinus rhythm. In particular, they exhibit lower vector amplitudes in all three dimensions and somewhat flatter 3‐dimensional loops, as compared to healthy subjects. These features could be utilized by statistical or machine learning predictive models to identify PAF from sinus rhythm electrocardiograms.

## 4600

### Deep Learning Predicts Myocardial Infarction Incidence From Multiple ECG Features

#### Fenella Downes ^1^, Riccardo Cavarra^1^, Shahid Aziz^2^, Shaheim Ogbomo‐Harmitt^1^, Oleg Aslanidi^1^

##### ^1^King's College London, United Kingdom, ^2^North Bristol NHS Trust, United Kingdom

**Introduction:** Myocardial infarction (MI) is a leading cause of cardiovascular mortality, with increasing global prevalence. The 12‐lead ECG is crucial for diagnosis, but manual interpretation is error‐prone and time‐consuming. Clinicians rely on the ST‐segment elevation to make a diagnosis, but this approach misses over one third of all MI cases. Recent studies suggest additional ECG features may enhance diagnostic accuracy.

**Methods:** This study evaluates whether deep learning (DL) models trained on full ECG features outperform models utilizing solely the ST‐segment during automated prediction of MI incidence. Gradient‐weighted class activation mapping (Grad‐CAM) is employed to identify key ECG regions influencing model predictions (see Figure). A residual network (ResNet) was trained to classify MI and healthy patients from 12‐lead ECG signals using a dataset comprising 2,050 healthy individuals and 2,043 MI patients (PTB‐XL database). An automated pipeline extracted ST segments from each lead. The dataset was split into 80% for training, 10% for validation, and 10% for testing.

**Results:** For the full ECG, ResNet achieved an accuracy of 90.00%, F1 score of 0.88, and AUC of 0.88. In contrast, when trained only on the ST‐segment, ResNet's performance decreased (accuracy: 85.85%, F1 score: 0.82, AUC: 0.84). Grad‐CAM analysis revealed that the full‐ECG model leveraged additional ECG features beyond the ST‐segment, namely the QRS complex. Further analysis on the latter showed a statistically higher entropy and increased QRS duration for MI subjects compared to healthy subjects.

**Conclusion:** Thus, our study highlights the importance of the QRS complex for MI detection by DL.

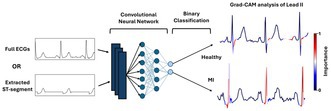

## 4614

### PowerECG: A Portable and Versatile ECG Analysis Platform for Rapid Cardiac Screening

#### Mehrdad Shahmohammadi Beni^1^, Derek Wu^2^, Hamza Waraich^3^, Quinncy Lee^4^, John Kwok Tai Chui^5^, Tong Liu^6^, Gary Tse ^1^

##### ^1^School of Nursing and Health Sciences, Hong Kong Metropolitan University, Hong Kong, China, ^2^McMaster University, Hamilton, Canada, ^3^Royal College of Surgeons in Ireland, Dublin, Ireland, ^4^PowerHealth Research Institute, Hong Kong, China, ^5^School of Science and Technology, Hong Kong Metropolitan University, China, ^6^Tianjin Institute of Cardiology, Second Hospital of Tianjin Medical University, China

**Introduction:** PowerECG offers a portable, easy‐to‐use graphical user interface (GUI) written in Python. It processes XML‐based ECG data, identifies key waveform features, and applies machine learning to analyze scanned ECG images, enabling rapid screening, reporting, and prognostic insights.

**Methods:** PowerECG is a Python‐based GUI tool built with Tkinter and specialized libraries for signal processing, data visualization, and YOLO‐based deep learning (Figure 1A). Its workflow involves reading ECG data from XML, detecting R‐peaks for RR intervals, calculating parameters (QT, QRS, ST segments), and recognizing ECG leads or waveforms from scanned images. The system also generates vectorcardiograms (VCGs) via the Dower inverse matrix, offers screening for Brugada pattern, and includes a voice output feature. PowerECG identifies J‐point elevations and ST/T changes, prolonged QT intervals, atrial fibrillation, sinus tachycardia or bradycardia, left ventricular hypertrophy, and premature ventricular contractions.

**Results:** PowerECG can accurately identify R‐peaks, measure interval durations (e.g., QT, QRS), and detect clinically relevant abnormalities such as ST‐segment elevation and T‐wave inversion. PowerECG can generate a sample ECG waveform plot (Figure 1B) on standard ECG paper and a vectorcardiogram (VCG) overlaying a 3D human heart model (Figure 1C). Comprehensive diagnostic reports can be produced in English, traditional and simplified Chinese, Spanish, and Japanese (Figure 1D). Using a YOLO‐based detection algorithm, distinct waveforms in each lead can be automatically identified (Figure 1E).

**Conclusion:** PowerECG is a versatile and user‐friendly ECG analysis tool that may improve clinical workflows by integrating automated screening and advanced visualization, making cardiac assessments more efficient for healthcare providers.

## 4618

### Conformal Prediction Improves Acute Myocardial Infarction Identification From 12‐Lead ECGs: A Practical Deep Learning Application

#### Dillon Dzikowicz ^1^, J.J. Garcia^2^, Jessica Zègre‐Hemsey^2^, Rebecca Kitzmiller^2^, Darlene Rogers^3^, Jason Betts^3^, Monique Bouvier^3^, Abhinav Goyal^3^, Xiao Hu^3^, Ran Xiao^3^

##### ^1^University of Rochester, New York, United States, ^2^University of North Carolina‐ Chapel Hill, North Carolina, United States, ^3^Emory University, Georgia, United States

**Introduction:** Early detection of acute myocardial infarction (AMI) is essential for timely reperfusion therapy and improved outcomes. Deep learning (DL) models applied to pre‐hospital electrocardiograms (ECGs) show promise by mapping ECG data to AMI risk probabilities. However, using fixed thresholds to classify risk may be insufficient in clinical settings with diagnostic uncertainty. Conformal prediction addresses this by assigning each prediction to one of three categories—high‐risk, low‐risk, or uncertain—based on probability bounds.

**Methods:** We implemented conformal prediction on a pre‐trained DL model developed using the PTB‐XL dataset (21,799 12‐lead ECGs with expert AMI labels). Three conformal methods—split conformal prediction (CP), class‐conditional conformal prediction (CCCP), and learn‐then‐test (LTT)—were applied. Thresholds were calibrated to ensure prediction error rates below 10%. The area under the receiver operating characteristic curve (AUC) was used to assess model performance. A non‐conformal prediction (NCP) version served as a baseline.

**Results:** AUCs were 0.923 (NCP), 0.932 (CP), 0.943 (CCCP), and 0.861 (LTT). Conformal methods improved discrimination but reduced sample coverage to 84.8% (CP), 86.7% (CCCP), and 77.3% (LTT), indicating that 13.3%–22.7% of predictions were flagged as uncertain.

**Discussion:** Conformal prediction enhances AMI detection by prioritizing confident predictions while deferring uncertain cases for further evaluation. This approach supports more reliable clinical decision‐making. Future work will refine uncertainty classification and explore deployment in pre‐hospital care settings.

## 4682

### Artificial Intelligence‐Enabled Sinus Electrocardiograms for the Detection of Paroxysmal Atrial Fibrillation Benchmarked Against the CHARGE‐AF Score

#### Constantine Tarabanis^1^, Vidya Koesmahargyo^2^, Robert Ronan^2^, Dimitrios Tachmatzidis^3^, Constantinos Bakogiannis ^3^, Vassilis Sousonis^4^, Stylianos Tzeis^4^, Vassilios Vassilikos^4^, Lior Jankelson ^2^

##### ^1^Division of Cardiovascular Medicine, Department of Medicine, Brigham and Women's Hospital, Harvard Medical School, Boston, MA, USA, ^2^Leon H. Charney Division of Cardiology, NYU Langone Health, New York University School of Medicine, New York, NY, USA, ^3^3rd Cardiology Department, Hippokrateion University Hospital, Aristotle University of Thessaloniki, Thessaloniki, Greece, ^4^Department of Cardiology, Mitera Hospital, Athens, Greece

**Introduction:** Atrial fibrillation (AF) is often underdiagnosed due to its episodic and asymptomatic nature. Early identification of at‐risk patients enables timely preventive strategies, such as anticoagulation, to reduce AF‐related complications. Artificial intelligence (AI) algorithms applied to sinus rhythm electrocardiograms (ECGs) show potential for latent AF detection, yet their added value over established scores like CHARGE‐AF remains unclear. This study aimed to develop and validate a convolutional neural network (CNN)‐based AI‐ECG model for paroxysmal AF detection and benchmark it against CHARGE‐AF.

**Methods:** We curated 157,192 sinus ECGs from 76,986 patients across the NYU Langone Health system, splitting them into training, validation, and test sets. External validation cohorts were drawn from two Greek hospitals: Ippokrateio (Thessaloniki) and Hygeia (Athens). A CNN using ECG time series data—alone and combined with CHARGE‐AF features—was trained to predict incident AF. Model performance was evaluated using AUC, AUPRC, sensitivity, specificity, and F1 score.

**Results:** In the NYU test cohort (*n* = 15,343), the ECG + CHARGE‐AF model achieved the highest performance: AUC 0.89 (95% CI: 0.88–0.89), AUPRC 0.69 (0.67–0.70). All AI‐ECG models outperformed CHARGE‐AF. In the Greek external cohort (*n* = 306), the same model achieved AUC 0.85 (0.81–0.88), AUPRC 0.78 (0.71–0.84), performing comparably to CHARGE‐AF. This reduced incremental gain in the external cohort was explainable through observed differences in CHARGE‐AF score distributions between positive and negative classes.

**Conclusions:** AI‐ECG can improve paroxysmal AF detection beyond CHARGE‐AF, though gains are influenced by the relative risk profile of positive and negative cases in the target population.

## 4582

### Comparative Outcomes of Ischemic and Non‐Ischemic Dilated Cardiomyopathy in ICD Recipients: A 30‐Year Retrospective Analysis

#### Themistoklis Pateromichelakis ^1^, Emmanouil Koutalas^1^, Eleutherios Kallergis^1^, Hercules Mavrakis^1^, Emmanouil Kanoupakis^1^, Georgios Kochiadakis^1^

##### ^1^University Hospital of Heraklion, Heraklion, Crete, Greece

**Background:** Ischemic cardiomyopathy (ICM) and non‐ischemic dilated cardiomyopathy (NIDCM) are the two main entities included in the landmark studies that established the use of implantable‐cardioverter‐defibrillators (ICDs). While arrhythmogenesis is in both cases triggered primarily in fibrotic areas of the ventricular myocardium due to reentry mechanisms, little is known regarding outcomes of patients with ICM versus NIDCM after the implantation of the ICD.

**Methods:** Data from the ICD registry of our Cardiology department were analyzed. The registry encompasses data from 1993 to the present. All patients that receive an ICD for primary or secondary prevention in our hospital are registered. We retrospectively compared the two main subgroups of patients, those with ICM and NIDCM regarding appropriate therapies from the ICD (anti‐tachycardia pacing or shock) during a mean follow‐up period of over 15 years.

**Results:** A total of 1582 patients were included in the analysis. Of them, 1064 suffered from ICM and the rest from NIDCM. 1265 patients received an ICD for primary prevention and 317 patients for secondary prevention. Regarding the likelihood of receiving appropriate therapy, no significant difference was observed between ICM and NIDCM patients overall. In the primary prevention subgroup, the incidence of appropriate therapy was similar between groups; however, NIDCM patients experienced a significantly shorter time to therapy (*p* = 0.002). In contrast, within the secondary prevention subgroup, while time to therapy was comparable between the two groups, NIDCM patients received appropriate therapies significantly more often than ICM patients (52.3% vs. 37.9%, *p* = 0.021).

**Conclusions:** While primary prevention outcomes are similar between ICM and NIDCM, patients with NIDCM may be diagnosed later in the disease course. In secondary prevention, the risk of malignant arrhythmias is significantly higher in the NIDCM subgroup compared to ICM. A potential genetic predisposition giving rise to more wild phenotypes in NIDCM, along with the evolving nature of its arrhythmogenic substrate, appears to play a crucial role in this increased risk.

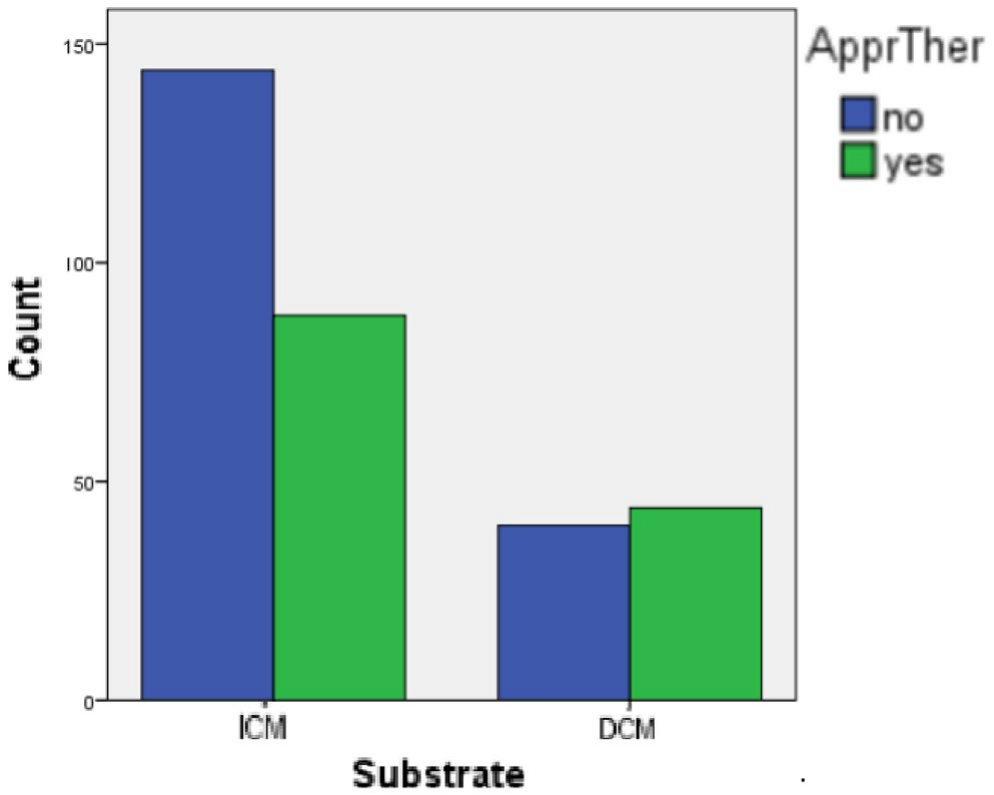

## 4584

### Preliminary Assessment of Cognitive Function in Atrial Fibrillation Patients Prior to Catheter Ablation Using the REMEDES for Alzheimer‐(R4Alz) Tool

#### Sotirios Chiotis ^1^, Georgios Giannopoulos^1^, Maria Toumpourleka^1^, Alexandros Evaggeliou^1^, Konstantinos Triantafyllou^1^, Vasileios Vassilikos^1^

##### ^1^Third University Department of Cardiology, “Hippokration” General Hospital, Aristotle University of Thessaloniki, Thessaloniki, Greece

**Background:** Cognitive impairment is prevalent in atrial fibrillation (AF) patients, linked to reduced cerebral perfusion, stroke risk, and microembolic events. While the Montreal Cognitive Assessment (MoCA) evaluates global cognitive function, REMEDES for Alzheimer (R4Alz), a recently developed tool, focuses on domains like working memory, attention control, and cognitive flexibility. This preliminary analysis aims to identify potential cognitive differences between AF patient subgroups and associations of cognitive performance with various clinical factors, using both MoCA and R4Alz tools, with plans to evaluate post‐ablation changes.

**Methods:** AF patients undergoing evaluation for CA completed cognitive assessments using MoCA and selected R4Alz subdomains. Baseline cognitive performance was compared across patient subgroups, and correlations between MoCA and R4Alz scores and relationships with clinical risk factors were examined.

**Results:** In 61 AF patients (41% female; 51% paroxysmal, 49% persistent; mean age 61.5 ± 10.6 years), MoCA and R4Alz total scores showed a moderate positive correlation (Spearman's rho = 0.33, *p* = 0.009). Among different subgroups, significant changes were found between patients with paroxysmal and persistent AF with the R4Alz (mean score 179.2 vs. 171.1, *p* = 0.005), while MoCA scores did not differ significantly (mean score 27.4 vs. 26.9, *p* = 0.3). R4Alz scores showed a significant negative correlation with CHA2DS2‐VASc (rho = −0.41, *p* < 0.001), while correlations with MoCA were non‐significant (rho = −0.08, *p* = 0.54).

**Conclusion:** R4Alz may effectively assess cognitive domains in AF patients, potentially identifying deficits overlooked by MoCA. Future analyses will examine follow‐up cognitive assessments post‐ablation to determine the impact of rhythm control on cognitive function.

## 4601

### Relationship of Beat‐to‐Beat P‐Wave Index to Left Atrial Low‐Voltage Areas in Patients With Paroxysmal Atrial Fibrillation

#### Dimitrios Tachmatzidis ^1^, Antigoni Sakellaropoulou^2^, Georgios Giannopoulos^1^, Konstantinos Letsas^2^, Antonios Antoniadis^1^, Dimitrios Asvestas^2^, Dimitrios Filos^3^, Panagiotis Mililis^2^, Michael Efremidis^2^, Ioanna Chouvarda^3^, Vassilios Vassilikos^1^

##### ^1^3rd Department of Cardiology, School of Medicine, Aristotle University of Thessaloniki, Greece, ^2^2nd Department of Cardiology, Laboratory of Cardiac Electrophysiology, Evangelismos General Hospital, Athens, Greece, ^3^Laboratory of Computing, Medical Informatics and Biomedical Imaging Technologies, School of Medicine, Aristotle University of Thessaloniki, Greece

**Introduction:** Atrial fibrillation (AF) is associated with left atrial (LA) fibrosis. The beat‐to‐beat (B2B) index—a non‐invasive marker derived from P‐wave morphology and wavelet analysis—has demonstrated predictive value for AF incidence and recurrence. This study investigates whether this index correlates with the extent of LA low‐voltage areas (LVAs).

**Material & Methods:** Patients with symptomatic paroxysmal AF without evident structural remodeling who were scheduled for pulmonary vein isolation were enrolled. High‐density voltage maps identified low‐voltage areas (LVAs < 0.5 mV), with patients stratified by small versus large LVAs. The B2B index, alongside conventional P‐wave characteristics such as P‐wave duration, was compared between these groups.

**Results:** Among the 35 included patients (87% male, median age 62 years), the median LVA was 7.7 cm^2^ (4.4–15.8), representing 5.6% (3.3–12.1) of the total LA endocardial surface. Patients with large LVAs demonstrated significantly higher B2B index values (0.65 [0.56‐0.77] vs. 0.57 [0.52‐0.59], *p* = 0.009) and longer P‐wave durations (146 ms [123‐165] vs. 135 ms [121‐141], *p* = 0.048). The B2B index had superior predictive value for large LVAs (c‐statistic 0.75, *p* = 0.006) compared to P‐wave duration (c‐statistic 0.70, *p* = 0.013). In multivariable adjustment, the first maintained independent predictive value (*p* = 0.04) while the later became non‐significant (*p* = 0.08), with the combined model achieving excellent discrimination (c‐statistic 0.81).

**Conclusions:** In patients with paroxysmal AF, B2B P‐wave analysis emerges as a valuable non‐invasive marker for detecting LA LVAs, reflecting underlying fibrosis. These findings provide a pathophysiological basis for the B2B index's potential clinical utility as a decision‐making tool.

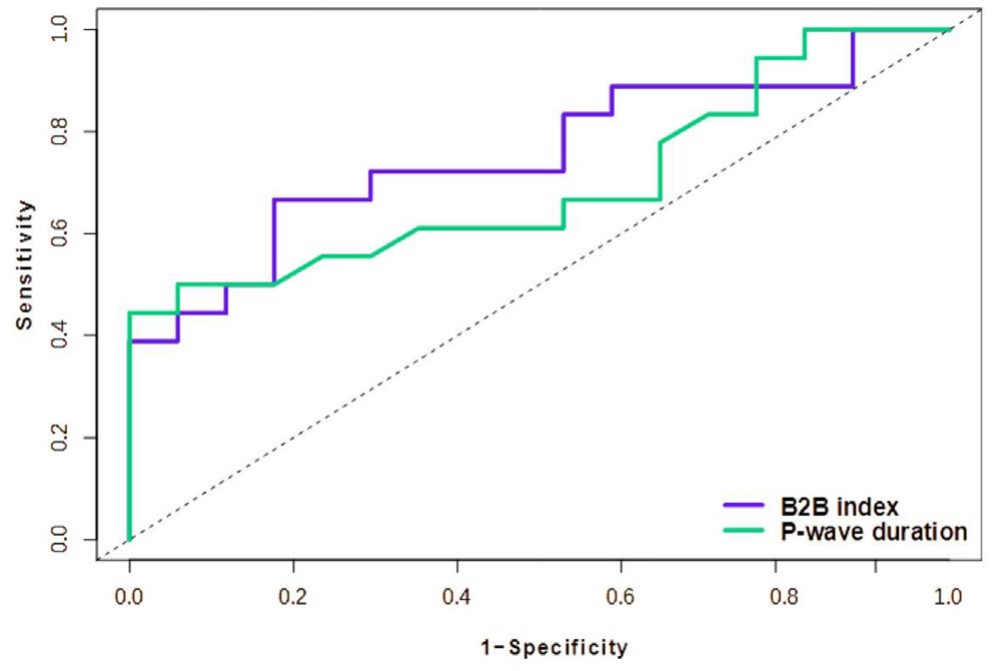

## 4681

### Reconstruction of 12‐Lead ECG From 3‐Lead Wearable Monitoring: A Clinically Validated Deep Neural Network Approach

#### Mikhail Chmelevsky^1^, Khamzin Khamzin^2^

##### ^1^Fondazione Cardiocentro Ticino, Lugano, Switzerland, ^2^Saint Petersburg State University, Saint‐Petersburg, Russia

**Background:** Remote cardiac monitoring through wearable devices is rapidly expanding but typically limited to 1–3 leads, reducing diagnostic power. This study aims to reconstruct full 12‐lead ECGs from only 3‐lead input using deep learning, enabling broader diagnostic capabilities from minimal sensors.

**Methods:** A convolutional neural network (CNN) and transformer‐based hybrid model were developed to synthesize 12‐lead ECG signals from three orthogonal leads (I, II, V2). Model training utilized a merged dataset from three large open‐access repositories: Chapman University, PTB‐XL, and Shandong Provincial Hospital totaling 41170 annotated 12‐lead ECG recordings. Data were split 80%:20% for training and validation. Label categories included normal ECGs, conduction blocks (LBBB, RBBB, etc.), ventricular preexcitation, and various myocardial infarctions. The reconstructed leads were compared to original signals using correlation coefficients (Corr), mean absolute error (MAE) and mean squared error (MSE) per lead. A prospective clinical study enrolled 100 patients undergoing real‐time 3‐lead ECG monitoring via a wearable device. The model‐reconstructed 12‐lead ECGs were compared against simultaneously recorded clinical standard 12‐lead ECGs, confirming model generalizability and clinical applicability.

**Results:** Correlation across all leads averaged 0.78 with highest agreement in leads I (0.99), V5 (0.84) and V6 (0.84). MAEs remained below 0.07 mV for most leads. Figure 1 illustrates the per‐lead distribution of these metrics.

**Conclusion:** Our approach demonstrates high‐fidelity reconstruction of 12‐lead ECGs from 3‐lead wearable data with robust generalization across datasets and real‐world clinical validation. This method has the potential to enhance remote diagnostics, especially for arrhythmia and ischemia detection while reducing hardware complexity.

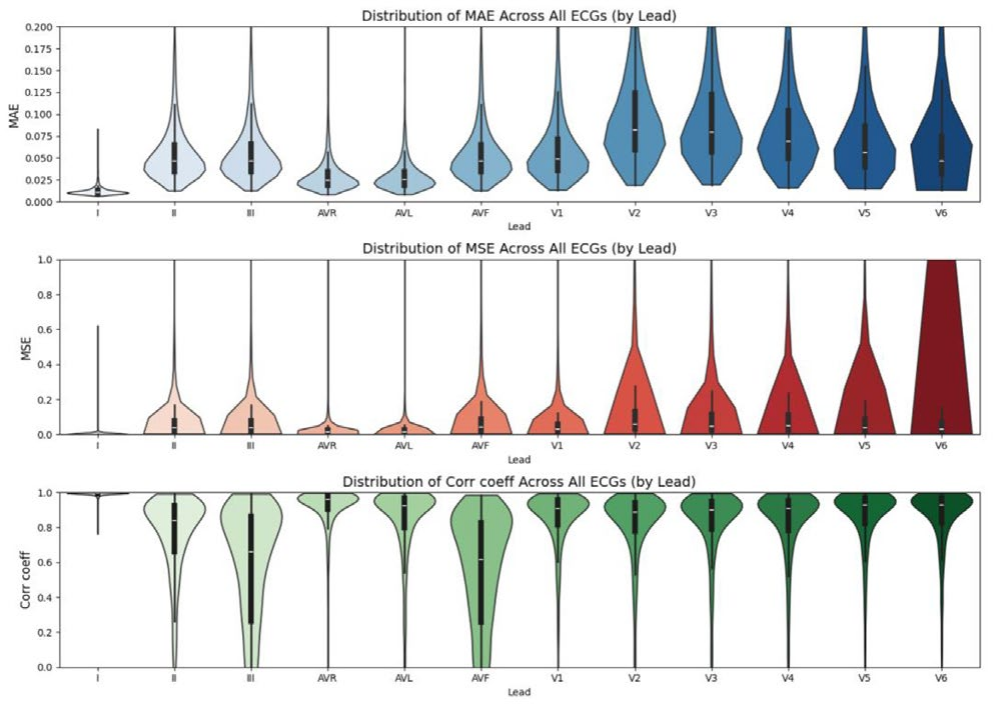

## 4581

### Pulsed‐Field Ablation for Long Standing Persistent Atrial Fibrillation: A Propensity Score Matched Comparison With Hybrid Ablation

#### Ioannis Doundoulakis ^1^, Domenico Giovanni Della Rocca^1^, Luigi Pannone^1^, Gezim Bala^1^, Alexandre Almorad^1^, Erwin Ströker^1^, Juan Sieira^1^, Dimitrios Tsiachris^2^, Serge Boveda^3^, Andrea Sarkozy^1^, Gian Battista Chierchia^1^, Carlo De Asmundis^1^

##### ^1^Heart Rhythm Management Centre, Postgraduate Program in Cardiac Electrophysiology and Pacing, University Hospital Brussels‐Free University Brussels, European Reference Networks Guard‐Heart, Brussels, Belgium, ^2^First Department of Cardiology, National and Kapodistrian University, “Hippokration” Hospital, Athens, Greece, ^3^Département de Rythmologie, Clinique Pasteur, Toulouse, France

**Introduction:** Rhythm control of long‐standing persistent AF is significantly more challenging, due to advanced electrical and structural remodeling of the left atrium. As such, the procedural endpoint for these patients remains doubtful. The aim of this study was to explore the 1‐year efficacy of pulsed field ablation (PFA) versus a single‐staged, radiofrequency‐based hybrid ablation strategy.

**Methods:** This is a prospective multicenter observational study. We included consecutive patients with long‐standing persistent AF (defined as continuous AF with duration > months) undergoing a first left atrium ablation with a PFA platform (Farapulse, Menlo Park, CA, USA) or monolateral, thoracoscopic hybrid radiofrequency ablation at three different European centers. Propensity score matching (1:1 ratio) was adopted to attenuate the imbalance in clinical characteristics.

**Results:** 55 consecutive PFA and 79 hybrid patients were included in the study. The mean age was 67.3 (±10) years, 38.1% were female and the mean CHA2DS2VASc score was 2.7 ± 1.4. Procedural durations was 99.7 ± 22.9 with PFA and 263 ± 61.3 min with hybrid (*p* < 0.001). Arrhythmia recurrence occurred in 17 (30.9%) PFA and 33 (41.8%) hybrid patients (*p* = 0.201). No difference in antiarrhythmic drug use was found (PFA *N* = 23, 40.4% vs. Hybrid *N* = 33, 41.8 %; *p* = 0.996). No significant differences were documented in the type of recurrent arrhythmia. Propensity score matching based on six clinical variables yielded 43 patients per group. Follow‐up results remain unchanged, with the 1‐year Kaplan–Meier analysis showing freedom from any atrial tachyarrhythmia in 69.8% of PFA and 53.5% of hybrid patients (log‐rank *p*‐value: 0.121). Major periprocedural complications occurred in 2 (3.6%) PFA and 12 (15.2%) hybrid patients. Two (3.6%) PFA patients with pre‐existing left ventricular systolic dysfunction (EF< 5%) required inotropic therapy due to acute heart failure with low cardiac output and hypotension (systolic blood pressure < 0 mmHg); hemodynamics normalized within 24 h. Among 12 (15.2%) complications in the hybrid group, three patients experienced acute respiratory insufficiency, one patient had a hemothorax, four experienced pneumothorax, and three patients had a pericarditis. One patient experienced a late tamponade (7 days after the procedure).

**Conclusion:** PFA contributed to significantly shorter procedural times and lower procedural complications. Long‐term arrhythmia freedom in patients with long‐standing persistent

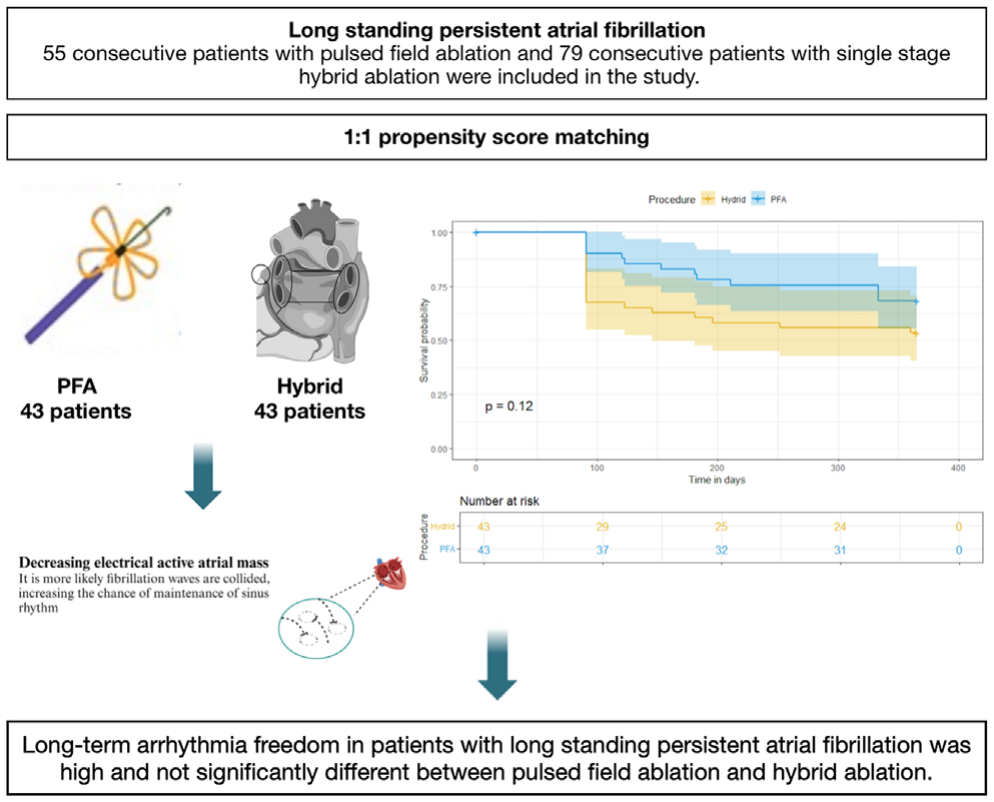

## 4587

### The Role of Multipoint Left Ventricular Pacing Compared To Optimized Cardiac Resynchronization Therapy on Long‐Term Follow‐Up in Chronic Heart Failure Patients

#### Polychronis Dilaveris ^1^, Panos Xydis^1^, Christos‐Konstantinos Antoniou^1^, Konstantinos Konstantinou^1^, Nikolaos Magkas^1^, Konstantinos Tsioufis^1^, Christina Chrysohou^1^, Panagiota Manolakou^1^

##### ^1^1st University Department of Cardiology, Hippokration Hospital, National & Kapodistrian University of Athens Medical School, Athens, Greece

**Introduction:** The aim of this study was to assess the capacity of optimized multipoint pacing (MPP) over cardiac resynchronization therapy (CRT), in terms of clinical, functional, and echocardiographic parameters among dyssynchronous heart failure (HF) patients on the 5‐year mortality.

**Methods:** We evaluated the echocardiographic and clinical response of 80 patients (Caucasian, 77.5% male, 68.4 ± 10.1 years, 53.8% ischemic cardiomyopathy) with HF under optimal medical treatment, treated with either CRT with hemodynamic and electrical optimization of the LV pacing site (optimized [OPT‐CRT] *n* = 34, 49%), or OPT combined with MPP (OPT‐MPP group, *n* = 36, 51%) for a follow‐up of 5 years.

**Results:** During the 5‐year follow‐up, 36 patients died (45 per 100 patients mortality rate). There was a significant difference in death rates between OPT (54%) and OPT‐MPP (40%), revealing that OPT‐MPP was related with 7% less probability of death in a 5‐year follow‐up, compared with OPT‐CRT (*p* = 0.01). The diseased patients had higher prevalence of ischemic HF, impaired baseline ventricle‐arterial coupling (VAC), higher baseline left atrial (LA) volume, impaired baseline LA strain and lower baseline right ventricle (RV) strain (all *p*‐values < 0.05). Furthermore, OPT‐MPP had higher survival rates, through beneficial effect on VAC (OR = 0.03, 95% CI 0.02–0.467) and LA strain (OR = 0.68, 95% CI 0.507–0.929), reflected on better score on Minnesota QoL questionnaire (OR = 0.89, 95% CI 0.799–0.99), compared to OPT‐CRT.

**Conclusions:** In chronic HF patients under CRT implantation, OPT‐MPP showed a beneficial effect on survival especially compared to OPT‐CRT only, through favorable changes in various clinical, functional, and echocardiographic parameters, as well as quality of life.

## 4606

### Utilization of Preprocedural Electrocardiographic Tracings by a Deep Machine Learning Model to Detect Atrial Fibrillation Recurrence After Catheter Ablation

#### Evangelia Myrovali ^1^, Georgios Giannopoulos^1^, Dimitrios Hristu‐Varsakelis^2^, Dimitrios Tachmatzidis^1^, Konstantinos Bakogiannis^1^, Vassilios Vassilikos^1^

##### ^1^3rd Cardiology Department, Aristotle University of Thessaloniki, Hippokration General Hospital, Thessaloniki, 54642, Greece, ^2^Department of Applied Informatics, University of Macedonia, Thessaloniki, 54006, Greece

**Background:** Catheter ablation for pulmonary vein isolation (PVI) is the most effective rhythm control treatment in patients with atrial fibrillation (AFib). However, considering that PVI is a procedure that is associated with a low but non‐zero risk of serious complications and 1‐year rates of sinus rhythm maintenance not exceeding 60%–70% in most studies, models that could reliably predict the risk of recurrence post‐PVI would be clinically useful. The aim of this work was to predict which patients are prone to recurrence after PVI by machine learning models, utilizing preoperative electrocardiographic (ECG) and clinical data.

**Methods:** We analyzed data from prospectively followed‐up patients undergoing PVI for paroxysmal AFib. We used pre‐procedural 5.9‐min ECG recordings in sinus rhythm (3 orthogonal leads, at 1000 Hz). We implemented a previously described deep machine learning model, specifically a convolutional neural network (CNN), applying window sliding as a data augmentation method, using a window of 4000 samples. For comparison, we assessed 44 clinical variables using a machine learning algorithm (shallow machine learning), specifically recursive feature elimination with XGBoost and 10‐fold cross‐validation.

**Results:** 123 patients scheduled for PVI were included. 52 had AFib recurrence within 1 year from the index procedure (42%). Among the initial 44 feature vectors inputted in the XGBoost model, left atrial volume and presence of metabolic syndrome were the most significant in terms of predictive value, achieving an accuracy of 0.70. On the other hand, the deep learning CNN achieved a higher classification accuracy of 0.82, utilizing only the preprocedural patient ECGs (see Table).

**Conclusion:** Deep learning artificial intelligence models may utilize the predictive information contained in ECGs and, thus, help in categorizing patients scheduled for PVI according to their risk for post‐ablation recurrence. This could be of use in personalizing decisions regarding PVI in patients with an indication for this procedure.

## 4608

### Harnessing Machine Learning for Accurate Ejection Fraction Classification in Echocardiographic Imaging

#### Dimitris Filos ^1^, Eleftheria Vorgiazidou^1^, Ioanna Chouvarda^1^

##### ^1^Laboratory of Computing, Medical Informatics and Biomedical Imaging Technologies, School of Medicine, Aristotle University of Thessaloniki, Thessaloniki, Greece

**Introduction:** Heart failure (HF) remains a major global health burden, driven by demographic shifts and cardiovascular risk factors. Ejection fraction (EF) is central to HF classification and treatment decisions, though echocardiographic assessment remains time‐intensive. Utilizing portable devices for bi/quadri‐chamber monitoring would benefit accurate EF estimation. This study introduces a machine learning (ML) approach employing radiomic features from echo imaging to classify patients into preserved (≥ 60%) and reduced EF (< 60%).

**Material & Methods:** Echo imaging four‐chamber planes from the CAMUS dataset, at end‐diastole (ED) and end‐systole (ES), were analyzed. Radiomic features were extracted from pericardial contours using predefined ultrasound‐optimized parameters. A multi‐stage, iterative feature selection process was applied utilizing different patient subsets, including statistical significance testing, correlation filtering, and Boruta analysis. Discriminative power was assessed via a composite score incorporating effect size, AUC, and overlap range across EF categories, selecting the top 10 features. An XGBoost classifier was trained with the top features in a 10‐fold partitioning strategy, using cross‐validation and grid search optimization for robust evaluation across diverse test sets.

**Results:** Feature selection identified 115 systematically selected features across 50 partitions, with 70% consistency. The top 10 features included wavelet‐, log‐sigma‐, and first‐order‐based texture metrics, primarily ES‐derived. The best‐performing XGBoost model achieved 93% accuracy on an independent cohort of 150 patients, correctly classifying 131/140 patients with preserved and 8/10 patients with reduced EF.

**Conclusion:** This radiomics‐based ML approach enables accurate EF classification via portable echocardiography, supporting timely HF diagnosis and management. Further advancement will involve ECG and clinical data integration.

## 4678

### Machine Learning‐Guided Differentiation of RVOT And LVOT Arrhythmias

#### Michail Botis ^1^, Dimitrios Tsiachris^1^, Georgios Botis^2^, Aristomenis Koumpanakis^2^, Lamprini Iro Bartsioka^1^, Ioannis Doundoulakis^1^, Athanasios Kordalis^1^, Christos Konstantinos Antoniou^1^, Aikaterini‐Eleftheria Karanikola^1^, George K. Matsopoulos^2^, Konstantinos Tsioufis^1^

##### ^1^First Department of Cardiology, Hippokration General Hospital, Athens, Greece, ^2^School of Electrical and Computer Engineering, Biomedical Engineering Laboratory, Athens, Greece

**Introduction:** Idiopathic premature ventricular complexes (PVCs) commonly originate from the right and left ventricular outflow tracts (RVOT and LVOT, respectively). The surface 12‐lead electrocardiogram (ECG) is commonly used to differentiate the anatomic site of origin, prior to catheter ablation. Machine learning methods can further enhance diagnostic accuracy.

**Materials & Methods:** In this study, ECG‐derived features, along with demographic characteristics, were analyzed from 43 patients (65.1% males, mean age 56 ± 16.6 years), who underwent successful catheter ablation of outflow tract‐originating idiopathic PVCs. The site of origin was depicted through 3‐D electroanatomical mapping. Several ML models, including Random Forest, Support Vector Machines (SVM), K‐Nearest Neighbors (KNN), and XGBoost, based on 65 ECG features, alongside patient age and gender, were trained to classify the site of origin as either RVOT or LVOT.

**Results:** XGBoost demonstrated the best classification performance, achieving a test accuracy of 0.90, with a sensitivity of 1.00 and a specificity of 0.80. The overall F1‐score was 0.91, indicating strong predictive ability. Feature importance analysis revealed that PVC QRS axis (importance: 0.1604), PVC Lead II S‐wave amplitude (0.1510), and PVC AVL Lead R‐wave amplitude (0.1338) were the three most significant predictors in distinguishing the arrhythmia origin.

**Conclusions:** These findings suggest that machine learning, particularly XGBoost, can effectively classify OTVAs based on ECG parameters, supporting its potential use in automated differentiation and pre‐procedural planning.

## 4629

### Arrhythmic Risk Stratification in Post‐Myocardial Infarction Patients With Preserved Ejection Fraction: Long‐Term Outcomes From the PRESERVE EF Study

#### Ioannis Doundoulakis ^1^, Dimitris Tsiachris^2^, Petros Arsenos^2^, Athanasios Kordalis^2^, Christos‐Konstantinos Antoniou^2^, Konstantinos Vlachos^3^, Stergios Soulaidopoulos^2^, Aggeliki Laina^2^, Emmanuel Kanoupakis^4^, Polychronis Dilaveris^2^, Theofilos M. Kolettis^5^, Konstantinos Trachanas^6^, Iosif Xenogiannis^7^, Panagiotis Korantzopoulos^5^, Skevos Sideris^6^, Nikolaos Fragakis^8^, Vassilios P. Vassilikos^9^, Konstantinos Tsioufis^2^, Konstantinos A. Gatzoulis^2^

##### ^1^Heart Rhythm Management Centre, Postgraduate Program in Cardiac Electrophysiology and Pacing, University Hospital Brussels‐Free University Brussels, European Reference Networks Guard‐Heart, Brussels, Belgium, ^2^First Department of Cardiology, National and Kapodistrian University, “Hippokration” Hospital, Athens, Greece, ^3^Institut Hospitalo‐Universitaire Institut des Maladies du Rythme Cardiaque, Electrophysiology and Heart Modeling Institute, Bordeaux, France; Haut‐Lévêque University Hospital, Bordeaux, France, ^4^Department of Cardiology, University Hospital of Heraklion, University of Crete, Heraklion, Crete, Greece, ^5^First Cardiology Division, University Hospital of Ioannina, University of Ioannina, Ioannina, Epirus, Greece, ^6^State Department of Cardiology, “Hippokration” General Hospital of Athens, Athens, Greece, ^7^Second Cardiology Department, National and Kapodistrian University of Athens, Attikon Hospital, Athens, Greece, ^8^Second Department of Cardiology, Hippokration General Hospital, Aristotle University of Thessaloniki, Thessaloniki, Greece, ^9^Third Cardiology Department, School of Medicine, Aristotle University of Thessaloniki, Hippokration General Hospital, Thessaloniki, Greece

**Introduction:** The PRESERVE EF study proposed a two‐step algorithm for risk stratification in post‐myocardial infarction patients with mid‐range and preserved left ventricular ejection fraction (LVEF). In the first step, patients with at least one non‐invasive risk factor (NIRF) were referred for electrophysiology study (second step) and were then considered for an implantable cardioverter‐defibrillator (ICD) in case of inducible malignant arrhythmia. This report presents the 8‐year follow‐up findings of the trial.

**Methods:** The primary endpoint was the occurrence of a major arrhythmic event (MAE), namely sustained ventricular tachycardia/fibrillation, appropriate ICD activation, or sudden cardiac death (SCD). We screened and included 575 consecutive patients (mean age 57 years, LVEF 50.8%). Of them, 204 (35.5%) had at least one positive NIRF. Forty‐one of 152 patients undergoing programmed ventricular stimulation (PVS) were inducible. Thirty‐seven (90.2%) of them received an ICD.

**Results:** Over a mean follow‐up of 106 ± 14.5 months, no SCDs were observed, while 12 ICDs (the major arrhythmic events prevalence in patients with ICD implantation reaching 29.3%) were appropriately activated. The updated (8.8‐year follow‐up) performance metrics of the proposed approach (included both steps with NIRFs and PVS) were as follows: Sensitivity = 100%, specificity = 94.8%, positive predictive value = 29.3%, and negative predictive value = 100%. Notably, no events occurred in patients with an LVEF > % who had not experienced STEMI.

**Conclusions:** Until a randomized trial provides a survival benefit in post‐myocardial infraction patients with preserved LVEF, the PRESERVE EF study remains the only evidence based risk assessment approach, despite its observational structure.

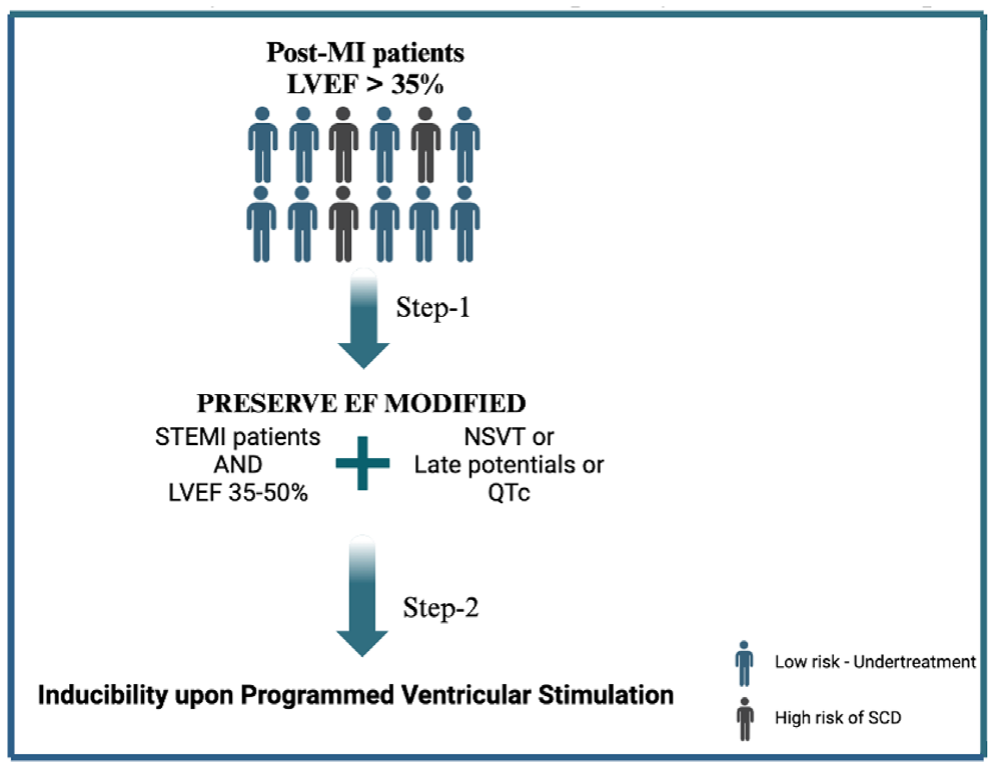

## 4651

### Atrial Myopathy in Hypertrophic Cardiomyopathy Patients Without a History of Atrial Fibrillation

#### Vaios Schismenos ^1^, Efstathios Pagourelias^1^, Sotirios Chiotis^1^, Orestis Pavlou^1^, Aristi Boulmpou^1^, Prokopis Mamolis^1^, Dimitrios Ntelios^1^, Christodoulos Papadopoulos^1^, Vasileios Vassilikos^1^

##### ^1^Third Cardiology Department, Hippokrateion University Hospital, Medical School, Aristotle University of Thessaloniki, Thessaloniki, Greece

**Background & Aim:** Left atrial (LA) volume index (LAVI), LA reservoir strain (LARS) and total atrial conduction time (TACT) (estimated by tissue Doppler imaging) are morphological and functional parameters reflecting LA structural and electrical remodeling (atrial myopathy). Aim of this study was to estimate correlations of atrial myopathy characteristics in a cohort of hypertrophic cardiomyopathy (HCM) patients without atrial fibrillation (AF) history.

**Methods:** We included 100 HCM patients (54 ± 21 years, 75% male, maximum wall thickness 19 ± 3.4 mm) without AF history who have consecutively undergone 2D‐speckle tracking echocardiography and cardiovascular magnetic resonance (CMR) with late gadolinium enhancement (LGE). TACT and LARS measurement are shown on the left panel. Burden of fibrosis (percentage of LV mass) was defined by LGE extent (>5 standard deviations compared to nulled myocardium) in CMR slices.

**Results:** Mean TACT was 140 ± 22 msec, with LAVI being 32 ± 16 mL/m^2^ and LARS 26% ± 14%. Among HCM demographic, phenotypic and functional characteristics tested, age and LV mass index were found to be the only independent regressors of TACT (*r* = 0.54, *p* < 0.0005 and *r* = 0.44, *p* = 0.002 respectively, right upper panel), while E/E′ (*r* = −0.44, *p* = 0.003) and fibrosis extent (*r* = 0.36, *p* = 0.02) were the strongest predictors of LARS and LAVI values respectively (right lower panel).

**Conclusions:** Atrial myopathy parameters seem to correlate with various morphological and functional characteristics of HCM. Atrial electro‐mechanical delay assessed through TDI‐based TACT correlates significantly with LVMI, whereas LARS and LAVI are strong regressors of elasticity properties, partially expressed through diastolic function and underlying fibrosis.

## 4610

### Optimizing Atrial Fibrillation Detection Through Hybrid ECG‐Based Feature Selection Using Extremely Randomized Trees and Non‐Linear Correlation Measures

#### Georgios Petmezas ^1^, Vasileios E. Papageorgiou^2^, Vassilios Vassilikos^1^, John A. Rogers^3^, Rod S. Passman^4^, Leandros Stefanopoulos^1^, Aggelos K. Katsaggelos^5^, Nicos Maglaveras^1^

##### ^1^School of Medicine, Aristotle University of Thessaloniki, Greece, ^2^Dept of Mathematics, Aristotle University of Thessaloniki, Greece, ^3^Dept of Material Science, Northwestern University, Evanston, IL, United States, ^4^Dept of Medicine, Northwestern University Feinberg School of Medicine, Chicago, IL, United States, ^5^Department of Electrical and Computer Engineering, Northwestern University, Evanston, IL, United States

**Introduction:** Atrial fibrillation (AFib) is the most prevalent abnormal heart rhythm, significantly increasing the risk of stroke and heart failure. Accurate and timely detection remains challenging, particularly due to the complexity of 12‐lead electrocardiogram (ECG) interpretation. While machine learning (ML) and deep learning (DL) models have demonstrated high accuracy in AFib detection, selecting the optimal input features is often non‐trivial.

**Material & Methods:** We propose a hybrid feature selection methodology that combines Extremely Randomized Trees (Extra‐Trees) with non‐linear correlation measures to identify the most discriminative ECG‐based features distinguishing AFib from normal sinus rhythm (NSR). Our analysis evaluates time‐based, entropy‐based and spectral hand‐crafted features extracted from 12‐lead ECG recordings of patients who underwent catheter ablation for AFib. Two novel metrics, the feature importance score (FIS) and the overall feature importance score (OFIS), are introduced to quantify feature relevance.

**Results:** The proposed feature selection approach demonstrated strong consistency between features derived from single‐lead and full 12‐lead analyses. The identified feature subset retained high discriminative power while significantly reducing dimensionality, ensuring robustness across different ECG leads.

**Conclusions:** This methodology can serve as a preprocessing step for ML/DL models, aiding clinicians in real‐time AFib detection during routine ECG screenings. By enhancing interpretability and reducing feature dimensionality, our approach offers a practical and reliable solution for improving diagnostic accuracy in electrophysiology.

## 4585

### Predictors of Arrhythmic Events in Hypertrophic Cardiomyopathy Patients With an Implantable Cardioverter‐Defibrillator: A Systematic Review and Meta‐Analysis

#### Sotirios Chiotis ^1^, Georgios Giannopoulos^1^, Ioannis Doundoulakis^2^, Aristi Boulmpou^1^, Vasileios Vassilikos^1^

##### ^1^Third University Department of Cardiology, “Hippokration” General Hospital, Aristotle University of Thessaloniki, Thessaloniki, Greece, ^2^Heart Rhythm Management Centre, Postgraduate Program in Cardiac Electrophysiology and Pacing, Universitair Ziekenhuis Brussel—Vrije Universiteit Brussel, European Reference Networks Guard‐Heart, Brussels, Belgium

**Background:** Hypertrophic cardiomyopathy (HCM) is a common genetic cardiac disorder and a leading cause of sudden cardiac death (SCD). Implantable cardioverter‐defibrillators (ICDs) are critical for SCD prevention, but risk stratification remains challenging. The objective of this study is to evaluate the predictive performance of conventional risk factors for arrhythmic events in HCM patients with ICDs.

**Methods:** We conducted a systematic search of PubMed, Cochrane Central Register of Controlled Trials (CENTRAL) and Clinical Trials from inception to November 2024, including studies reporting hazard ratios (HRs) for clinical, electrocardiographic, and imaging predictors of arrhythmic events in ICD recipients with HCM. Pooled HRs were calculated using a random‐effects model.

**Results:** 12 studies of 3,297 HCM patients with ICDs (91% primary prevention, 9% secondary prevention) were included, with a mean age of 50 years. The annual arrhythmic event rate was 5% (95% CI: 4%–7%) during a mean follow‐up of 4 years. Significant predictors of arrhythmic events included non‐sustained ventricular tachycardia (NSVT) (HR: 2.19, 95% CI: 1.62–2.98), left ventricular ejection fraction (LVEF) < 0% (HR: 1.91, 95% CI: 1.27–2.89), intraventricular pressure gradient (IVPG)> mmHg (HR: 1.92, 95% CI: 1.03–3.56), and secondary prevention indication (HR: 2.18, 95% CI: 1.39–3.41). Conversely, traditional risk factors such as extreme hypertrophy, family history of SCD, and syncope showed limited predictive value.

**Conclusion:** This analysis demonstrates that conventional markers like NSVT, LVEF < 0%, and IVPG> mmHg remain strong predictors of arrhythmic events in HCM patients with ICDs, but other traditional risk factors may lack predictive utility.

## 4595

### Assessment of Incidence of Fragmented QRS in the Standard Electrocardiogram in Patients With Acute Pulmonary Embolism

#### Piotr Bienias ^1^, Olga Dzikowska‐Diduch^1^, Tomasz Cader^1^, Szymon Staneta^1^, Katarzyna Dąbrowska^1^, Aisha Ou‐Pokrzewińska^1^, Michał Ciurzyński^1^, Piotr Pruszczyk^1^

##### ^1^Department of Internal Medicine and Cardiology with Center for Diagnostics and Treatment of Venous Thromboembolism, Medical University of Warsaw, Warsaw, Poland

**Introduction:** Various ECG abnormalities are often observed in acute pulmonary embolism (APE). Although ECG is not a sufficient tool for the diagnosis of APE, it may potentially contribute to improving the prognosis assessment in addition to established predictors. The aim of our study was to assess the occurrence of fragmented QRS complexes (fQRS) in APE, which are described not only as being associated with myocardial scarring or fibrosis, but also with conduction disturbances and depolarization.

**Material & Methods:** In our specialized center we examined 560 patients with confirmed APE. Except routine procedures, ECG was performed immediately after admission to the hospital. fQRS assessment was performed manually according to defined by Das et al. criteria. Subjects with ventricular pacing rhythm (*n* = 7) were excluded for further analysis. For each patients the simplified Pulmonary Embolism Severity Index was calculated. Assessment of APE‐related mortality risk (low, intermediate, high) was established due to ESC Guidelines.

**Results:** Ultimately, 553 patients aged 62.3 ± 18.9 years were included in the study (53.3% women). Sinus rhythm was present in 511 (92.4%), and AF/AFL in 42 (7.6%) patients. Completely normal ECG was found in 133 (24.0%), while ECG with any various abnormalities was found in 420 (76.0%) patients. Detailed results of fQRS occurrence in the studied subgroups are presented in the table. In some patients, different fQRS patterns co‐occurred.

**Conclusions:** Our results confirmed previous observations indicating a high incidence of fQRS in APE; however, in our group, it was not associated with the disease severity and the early mortality risk.

## 4624

### A Novel Variational Autoencoder Framework for Explainable AI‐ECG

#### Konstantinos Patlatzoglou ^1^, Libor Pastika^1^, Joseph Barker^1^, Ewa Sieliwonczyk^2^, Gul Rukh Khattak^1^, Boroumand Zeidaabadi^1^, Antônio H. Ribeiro^3^, James S. Ware^1^, Nicholas S. Peters^1^, Antonio Luiz P. Ribeiro^4^, Daniel B. Kramer^5^, Jonathan W. Waks^5^, Arunashis Sau^1^, Fu Siong Ng^1^

##### ^1^Imperial College London, England, United Kingdom, ^2^University of Antwerp and Antwerp University Hospital, Edegem, Belgium, ^3^Uppsala University, Uppsala, Sweden, ^4^Universidade Federal de Minas Gerais, Belo Horizonte, Brazil, ^5^Beth Israel Deaconess Medical Center, Boston, United States

**Introduction:** Artificial intelligence‐enhanced electrocardiogram (AI‐ECG) models have shown outstanding performance in a vast range of diagnostic and prognostic tasks, yet their black‐box nature hampers clinical adoption. Meanwhile, a growing demand for explainable AI (XAI) in medicine underscores the need for transparent, trustworthy decision‐making. Post‐hoc explainability techniques have largely fallen short, yielding ambiguous, inconsistent, and biased interpretations of model behavior.

**Methods:** In this work, we propose an inherently explainable framework using variational autoencoders (VAEs)—a technique which enables the discovery of structured and continuous axes of clinically interpretable ECG features. Specifically, we trained a convolutional β‐VAE model on over 3 million ECGs using primary and secondary care clinical datasets (BIDMC, TNMG). We next integrated a symbol‐concept association network (SCAN), allowing us to model bi‐directional associations between ECG features and clinical factors, while preserving the causal mechanisms used for decoding and explainability.

**Results:** The VAE model utilized up to 80 ECG features (latent factors) under maximal encoding capacity (Pearson's *R* = 1, 0.006 mV per sample MAE). Based on these features, SCAN was able to learn sparse associations with a limited number of ECG‐label pairs, when trained under clinical factors such as age, sex, and mortality risk. The model's decoding performance reached an MAE of 10.76 for age, an AUC of 0.81 for sex, and an AUC of 0.72 for 5‐year mortality risk. Population‐level interpretability was acquired by visualization of clinical factor traversals, which revealed both known and novel, nuanced ECG signatures—supporting the model's validity for sample‐ and model‐level explainability.

**Conclusions:** Our results demonstrate a state‐of‐the‐art VAE model and a novel framework (VAE‐SCAN) for explainable AI‐ECG. This approach has implications for biomarker discovery and the development of explainable, personalized, and population‐level clinical tools.

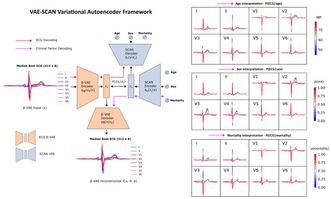

## 4633

### Feasibility and Early Outcomes of Conduction System Pacing in Heart Failure Patients With Left Bundle Branch Block

#### Polychronis Dilaveris ^1^, Vasiliki‐Chara Mystakidi^1^, Panteleimon Pantelidis^1^, Michael Spartalis^1^, Nektarios Souvaliotis^1^, Evangelos Oikonomou^1^, Gerasimos Siasos^1^

##### ^1^3rd University Department of Cardiology, NKUA, “Sotiria” hospital, Athens, Greece

**Background:** Conduction system pacing (CSP), particularly left bundle branch [area] pacing (LBB[A]P), is emerging as a physiological alternative to biventricular pacing in patients with heart failure and left bundle branch block (LBBB). In this study, we evaluated the feasibility and early electrical outcomes of CSP in a real‐world sample of heart failure patients.

**Case series:** We evaluated 15 consecutive patients (mean age: 68 ± 9 years; 47% female) who underwent CSP using a stylet‐driven system (Selectra 3D sheath, Biotronik, Berlin, Germany). Indications included low ejection fraction (EF) with intrinsic or pacing‐induced LBBB (*n* = 13) or failed conventional CRT (*n* = 2). An atrial lead was implanted in 13 patients; 2 had permanent atrial fibrillation. All received a right ventricular ICD lead if not previously present. Pacing mode was set to LBB or LBBAP for all patients. Pre‐implantation QRS duration averaged 162 ± 24 ms, which narrowed significantly post‐implantation to 108 ± 20 ms. Procedural success was 100% with no immediate complications. CSP resulted in improved electrical resynchronization. Device parameters were stable at early follow‐up.

**Conclusion:** CSP using a stylet‐driven system is feasible, safe, and results in significant QRS narrowing in heart failure patients with LBBB. It represents a promising alternative in cases of failed CRT or when LBBAP is preferred over traditional biventricular pacing.

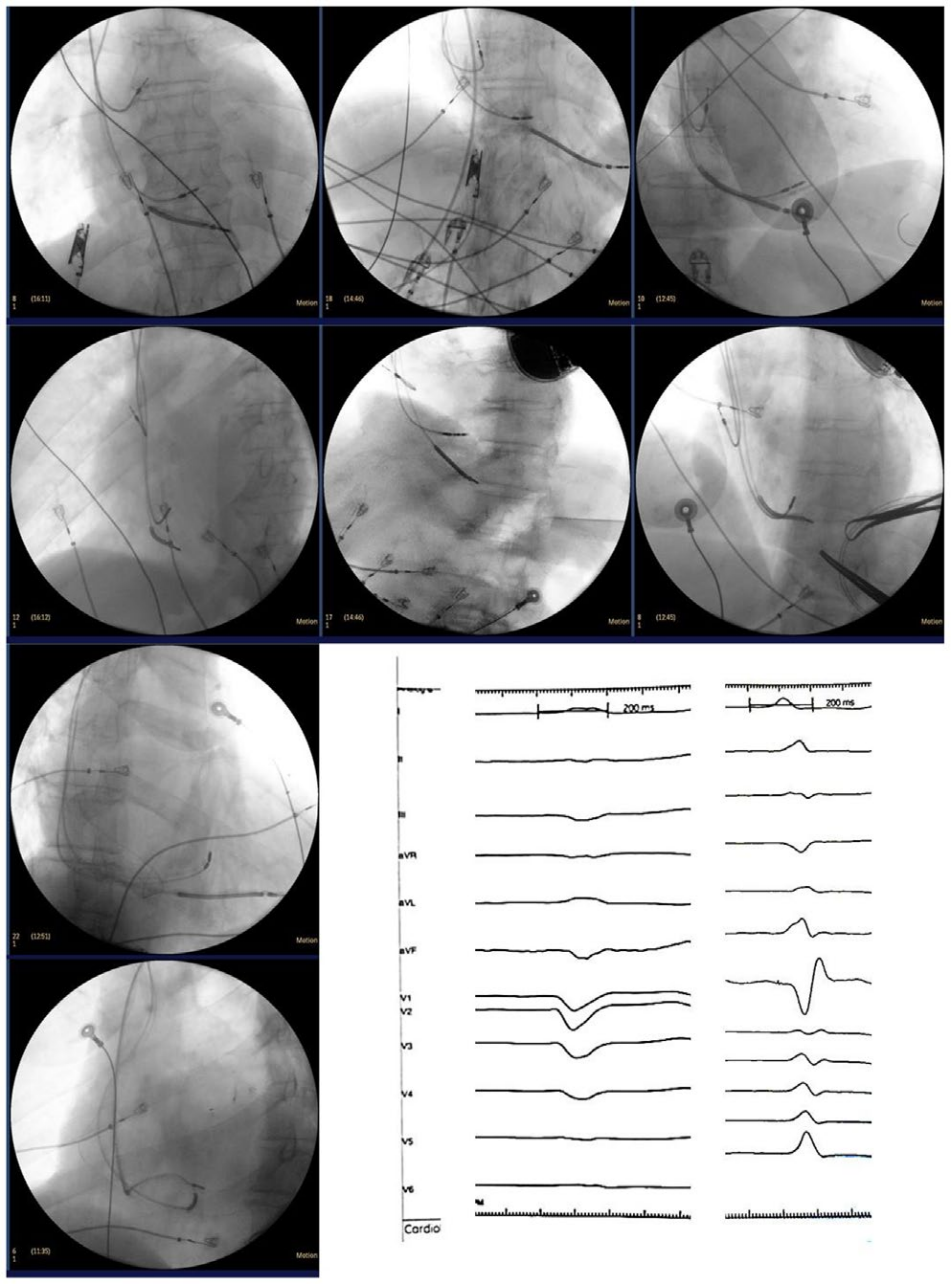

## 4592

### Meta‐Analysis on the Diagnostic Performance of AI in ECG for Hyperkalemia Detection

#### Antonios Keramas ^1,2^, Matthaios Didagelos^2^, Sophia Tsokkou^1^, Theodora Papamitsou^1^, Antonios Ziakas^1,2^

##### ^1^Laboratory of Histology‐Embryology, School of Medicine, Faculty of Health Sciences, Aristotle University of Thessaloniki, Thessaloniki, Greece, ^2^1st Cardiology Department, School of Medicine, Faculty of Health Sciences, AHEPA University General Hospital, Aristotle University of Thessaloniki, Thessaloniki, Greece

**Aim:** Current advances in Artificial Intelligence (AI) have allowed the development of deep learning algorithms that can detect hyperkalemia using ECG tracings. The current meta‐analysis aims to evaluate the sensitivity, specificity, and diagnostic accuracy of different AI models used for hyperkalemia detection.

**Materials and Methods**: A systematic search was conducted in PubMed, Scopus, Web of Science, Embase, IEEE Xplore, and arXiv to identify eligible studies up to March 2025. Keywords used included: “Artificial Intelligence” “ECG” and “Hyperkalemia” Overall, nine studies fulfilled the inclusion criteria. The meta‐analysis was conducted using a random effects model in R software.

**Results:** The pooled sensitivity of the AI models was 0.86 (95% CI: 0.80–0.91), with heterogeneity (*I* ^2^ = 69.1%), and specificity was 0.75 (95% CI: 0.68–0.81), with heterogeneity (*I* ^2^ = 77.7%). The average AUC across studies was above 0.88 (95% CI: 0.84–0.91), indicating high overall diagnostic performance and low heterogeneity (*I* ^2^ = 28.2%). The findings suggest that AI models can detect hyperkalemia with a high degree of reliability and consistency. The odds ratios showed DOR of 1.87 (95% CI: 0.82–4.25), suggesting heterogeneity in diagnostic performance between studies. Heterogeneity was very high (*I* ^2^ = 98.9%), indicating differences in population, ECG lead placement, or AI model type.

**Conclusion:** The findings indicate that AI models demonstrate a powerful tool for detecting hyperkalemia with high reliability and consistency. Implementing those models in medical care facilities could assist doctors with early hyperkalemiadiagnosis. However, further studies are needed to validate the results in different clinical settings and ECG equipment.

## 4594

### Wearable Devices for Quantifying Atrial Fibrillation Burden. A Systematic Review and Bayesian Meta‐Analysis

#### Ioannis Anagnostopoulos ^1^, Vrachatis Dimitrios^1^, Kousta Maria^1^, Giotaki Sotiria^1^, Katsoulotou Dimitra^2^, Karavasilis Christos^2^, Deftereos Gerasimos^1^, Avramides Dimitrios^2^, Giannopoulos Georgios^3^, Papaioannou Theodore^4^, Deftereos Spyridon^1^

##### ^1^Department of Interventional Cardiology and Electrophysiology, Evgenidio Hospital, Athens, Greece, ^2^Cardiology Department, Athens General Hospital “G. Gennimatas”, Athens, Greece, ^3^3rd Department of Cardiology, Aristotle University of Thessaloniki, Thessaloniki, Greece, ^4^Department of Biomedical Engineering, Medical School, National and Kapodistrian University of Athens, Greece

**Introduction:** Atrial fibrillation (AF) is the most common supraventricular arrhythmia and is associated with an impaired prognosis. Studies using implantable cardiac monitors suggest that this association is closely linked to AF burden, defined as the percentage of time spent in AF. Consequently, there is a growing need for affordable and comfortable alternative devices, such as wearables, capable of reliably monitoring AF burden in patients with AF.

**Material and Methods:** Major electronic databases were searched for studies comparing AF burden quantification using wearables and reference ECG monitoring methods. A Bayesian approach was adopted for the final analysis.

**Results:** Six studies including a total of 448 patients and 36978 h of valid simultaneous recordings, were analyzed. Bayesian analysis revealed no statistically significant differences between wearables and reference methods in AF burden quantification. The mean error was 1% (95% CrIs: −4% to 7%, Figure). Similar findings were observed in the subgroup analysis of studies assessing only smartwatches. Between study heterogeneity was low, and no evidence of publication bias was detected.

**Conclusions:** Our analysis suggests that AF burden quantification using wearables is comparable to reference ECG monitoring methods. These findings support the potential role of wearables in clinical practice, particularly for research and prognostic purposes. However, more studies are needed to determine whether the observed statistical equivalence translates to clinical significance, thereby supporting the widespread use of wearables in the assessment of rhythm control therapeutic strategies.

## 4599

### High‐Frequency Spectral Entropy: Novel Metric for Non‐Invasive Assessment of Left Atrial Fibrosis From Single‐Lead Electrocardiogram

#### Shaheim Ogbomo‐Harmitt^1^, Oleg Aslanidi^1^

##### ^1^King's College London, London, United Kingdom

**Introduction:** Atrial fibrillation (AF) is the most prevalent cardiac arrhythmia worldwide, significantly increasing the risk of stroke, heart failure, and dementia. Left atrial (LA) fibrosis has a critical effect on AF progression and treatment outcomes. Currently, cardiac magnetic resonance (CMR) imaging is the only non‐invasive method for assessing LA fibrosis, but its high cost and limited accessibility restrict widespread use. Electrocardiogram (ECG) is an affordable and widely available tool for evaluating cardiac function and characteristics. We propose a novel ECG‐derived metric, high‐frequency spectral entropy (HFSE), to assess the extent of LA fibrosis.

**Methods:** In‐silico simulations were conducted using 90 LA mesh models derived from CMR images. An additional 67 models with augmented fibrotic content were generated to increase the dataset. Atrial activity was simulated by solving the monodomain equation with the Courtemanche‐Ramirez‐Nattel cellular model. Lead II P‐waves were computed after registering each LA model to an idealized torso using the infinite volume conductor method. HFSE was computed by modifying the Shannon entropy of the power spectrum (spectral entropy) to emphasize higher frequencies through weighting.

**Results:** HFSE of the P‐wave correlates with the distribution of fibrotic tissue, with higher HFSE being characteristic of a greater degree of dense fibrotic tissue (see Figure). Results demonstrate a strong positive correlation (*r* = 0.66, *p* = 2.8e‐21) between lead II P‐wave HFSE and the percentage of dense LA fibrosis.

**Conclusion:** This study presents a novel non‐invasive ECG‐based metric for assessing LA fibrosis, offering a potentially cost‐effective and scalable alternative to the current imaging state‐of‐the‐art.

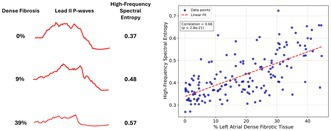

## 4609

### Detection of Myocardial Infarction Using Beat‐To‐Beat Analysis of 12‐Lead ECG

#### Spyridon Nikas ^1^, Anthi Chatziioannou^2^, Dimitrios Filos^3^

##### ^1^Radiology Department, General Hospital of Thessaloniki Papageorgiou, 546 29 Thessaloniki, Greece, ^2^Radiology Department, General Hospital of Thessaloniki Georgios Papanikolaou, 570 10 Thessaloniki, Greece, ^3^Laboratory of Computing, Medical Informatics and Biomedical Imaging Technologies, School of Medicine, Aristotle University of Thessaloniki, 541 24 Thessaloniki, Greece

**Introduction:** Myocardial Infarction (MI), a life‐threatening consequence of severe coronary artery disease, can be diagnosed using ECG. Rapid diagnosis and treatment are crucial, but interpretation requires expertise since some MIs show non‐specific findings. This work aims to develop a classification model to detect MI in ECG signals and distinguish them from healthy cases.

**Material & Methods:** A sample dataset of 415 patients (MI: 56, Healthy: 359) from PhysioNet's PTB‐XL database was used. Preprocessing reduced noise and detected ECG fiducial points. Time and time‐frequency domain features were extracted, followed by feature selection: low variance removal, statistical significance testing (*t* test with Bonferroni correction), elimination of highly correlated features (c > 0.8), and Recursive Feature Elimination. PCA reduced dimensionality, and features were normalized to [0,1] using min‐max scaling. A Random Forest (RF) classifier (1000 trees, 10‐fold cross‐validation) was trained, with SMOTE applied to address class imbalance.

**Results:** Among the 37 significant features, the top‐6 included lower T‐wave and higher S‐wave amplitudes in aVF, correlating with ST elevation in IMI, and higher T‐wave in aVL, associated with AMI. A higher synchronized QRS duration indicates severe ischemia and increased risk of fatal arrhythmias. Regarding model performance, the RF classifier achieved a balanced accuracy of 0.99, an F1 score of 0.95 and an AUC of 0.992.

**Conclusions:** Beat‐to‐beat analysis of the 12‐lead ECG led to development of a classification model that accurately distinguishes MI patients from healthy subjects. Further analysis for different MI types can improve the model's explainability.

## 4626

### Impact of Catheter Ablation Timing on Age‐Stratified Atrial Fibrillation Recurrence and Clinical Outcomes: A Meta‐Analysis

#### Karakasis Paschalis ^1^, Paschalis Karakasis^1^, Stylianos Tzeis^2^, Konstantinos Pamporis^3^, Panagiotis Theofilis^3^, Nikias Milaras^3^, Dimitrios Tsiachris^3^, Efremidis Michael^4^, Antoniadis Antonios^1^, Fragakis Nikolaos^1^

##### ^1^Second Department of Cardiology, Aristotle University of Thessaloniki, Hippokration General Hospital, Thessaloniki, Greece, ^2^Department of Cardiology, Mitera Hospital, 6, Erythrou Stavrou Str. 151 23, Marousi, Athens, Greece, ^3^First Department of Cardiology, School of Medicine, National and Kapodistrian University of Athens, Hippokration General Hospital, Athens, Greece, ^4^Department of Cardiology, Onassis Cardiac Surgery Center, Athens, Greece

**Introduction:** Catheter ablation is a well‐established treatment for symptomatic paroxysmal (PAF) or persistent atrial fibrillation (PsAF) refractory to antiarrhythmic agents, and current guidelines have also upgraded its role as a first‐line option for recurrent PAF. However, the optimal timing to maximize rhythm outcomes remains uncertain. To address this gap, the present study sought to investigate the association between diagnosis‐to‐ablation time (DAT) and age‐stratified atrial fibrillation (AF) recurrence and clinical outcomes.

**Method and Results:** Medline, the Cochrane Library, and Scopus were searched through February 18, 2025. Triple‐independent selection, extraction, and quality assessment were conducted, with evidence pooled via random‐effects meta‐analyses. Among 28 studies (41,431 participants) with a median 24‐month follow‐up, early ablation (DAT ≤ 1 year) significantly reduced AF recurrence compared to delayed ablation (hazard ratio [HR] 0.65, 95% CI 0.59–0.73). The benefit of early ablation was consistent for both PAF (HR 0.72, 95% CI 0.67–0.77) and PsAF (HR 0.70, 95% CI 0.61–0.81). Age‐stratified analysis revealed that this effect was significant regardless of age, with the greatest risk reduction observed in individuals ≤ 55 years (HR 0.49, 95% CI 0.34–0.71). Early ablation was also associated with a reduced risk of repeat ablation, new cardioversion, and cardiovascular hospitalization compared to delayed ablation. Higher CHA₂DS₂‐VASc scores, heart failure prevalence, and lower mean left ventricular ejection fraction were associated with greater benefits from early ablation.

**Conclusion:** Early catheter ablation within 1 year of AF diagnosis is associated with a lower risk of recurrence in both paroxysmal and persistent AF, with the strongest association observed in patients ≤ 55 years.

## 4627

### Clonal Hematopoiesis of Indeterminate Potential and Risk of Atrial Fibrillation: A Meta‐Analysis

#### Paschalis Karakasis ^1^, Eleftheria Lefkou^2^, Dimitrios Patoulias^3^, Nikolaos Fragakis^1^

##### ^1^Second Department of Cardiology, Aristotle University of Thessaloniki, Hippokration General Hospital, Thessaloniki, Greece, ^2^Blood Transfusion Unit, Medical School, University of Thessaly, Volos, Greece, ^3^Second Propedeutic Department of Internal Medicine, Faculty of Medicine, School of Health Sciences Aristotle, University of Thessaloniki, Greece

**Background and Aims:** Clonal hematopoiesis of indeterminate potential (CHIP) has recently been recognized as a significant risk factor for various non‐hematologic conditions, particularly cardiovascular diseases. However, the relationship between CHIP and atrial fibrillation (AF) remains underexplored to date. Given the conflicting findings in recent studies, the present meta‐analysis aimed to assess the association between CHIP and the incidence or recurrence of AF.

**Methods:** Medline, Cochrane Library and Scopus were searched until October 27, 2024. Triple‐independent study selection, data extraction and quality assessment were performed. Evidence was pooled using restricted maximum likelihood random‐effects meta‐analysis.

**Results:** Four studies comprising a total of 801,085 participants were included. Over a median follow‐up period of 9 years, participants with any CHIP (variant allele fraction [VAF] ≥ 2%) exhibited a significantly elevated risk of developing incident or recurrent AF compared to those in the non‐CHIP group (hazard ratio [HR] = 1.12; 95% confidence interval [CI] = [0.07 to 1.17], *p* < 0.0001; *I* ^2^ = 3%, heterogeneity *p* = 0.38). The presence of any CHIP was associated with a markedly increased risk for both incident and recurrent AF when these outcomes were analyzed separately. Furthermore, large CHIP (VAF ≥ 10%) was correlated with a heightened risk of incident AF, suggesting a potential dose‐response relationship. Leave‐one‐out sensitivity analyses identified no evidence of outliers.

**Conclusions:** CHIP is associated with increased risk of incident or recurrent AF. Further research is required to clarify the mechanisms underlying the observed association and to explore interventions aimed at mitigating this risk.

## 4647

### Pre‐Existing and New‐Onset AF on All‐Cause Mortality in Patients Undergoing TAVI‐Meta‐Analysis Study

#### Athanasios Saplaouras ^1^, George Bazoukis^2^, Panagiotis Mililis^1^, Ourania Kariki^1^, Stylianos Dragasis^1^, Stavroula Koskina^1^, Theodoros Efremidis^1^, Athanasios Makris^1^, Konstantinos Letsas^1^, Michael Efremidis^1^

##### ^1^Arrhythmia Unit, Onassis Cardiac Surgery Center, Athens, Greece, ^2^Department of Cardiology, Larnaca General Hospital, Larnaca, Cyprus

**Introduction:** Atrial fibrillation (AF) is a common comorbidity in patients with severe AS who are planning to undergo TAVI, while new‐onset AF can complicate the clinical course of these patients. This meta‐analysis aims to evaluate the impact of pre‐existing and new‐onset AF on all‐cause mortality in patients undergoing TAVI.

**Methods:** This meta‐analysis has been prepared in adherence to the Preferred Reporting Items for Systematic Review and Meta‐Analyses (PRISMA) guidelines.

**Results:** Finally, 30 studies provided data regarding the association between pre‐existing (18 studies) and/or new‐onset AF (12 studies) with all‐cause mortality and were therefore included in the quantitative analysis. Patients with new‐onset AF had a 67% higher risk of all‐cause mortality compared to patients without new‐onset AF (HR: 1.67, 95% CI [1.39–2.00], *p* < 0.001, *I* ^2^ 72%). Similarly, patients with pre‐existing AF had a 67% higher risk of all‐cause mortality compared to patients without pre‐existing AF (HR: 1.64, 95% CI [1.53–1.77], *p* < 0.001, *I* ^2^ 28%).

**Conclusions:** Pre‐existing and new‐onset AF is associated with an increased risk of all‐cause mortality in patients undergoing TAVI procedures. Further research is needed to evaluate the beneficial role of sinus rhythm maintenance and antiarrhythmic medication, especially in patients who develop new‐onset AF.

## 4630

### Non‐Invasive Electrocardiographic Risk Factors for Sudden Cardiac Death in Patients With ND‐LVC

#### Nikias Milaras ^1^, Konstantinos Gatzoulis^1^, Skevos Sideris^1^

##### ^1^“Hippokration” General Hospital of Athens, Athens, Greece

**Background:** Sudden cardiac death (SCD) remains a significant concern in patients with Non Dilated Left Ventricular Cardiomyopathy (ND‐LVC). Risk stratification for major arrhythmic events remains largely unknown for this population, whose entity was recently conceived.

**Objective:** This prospective study aimed to evaluate 60 ND LVC patients through cardiac MRI, Late potentials and several electrocardiographic risk factors deducted from an 24 h Holter monitor, and correlate them with adverse arrhythmic outcomes (VT/VF), with the goal of informing strategies for continuous risk stratification.

**Methods:** In this study, 60 patients with ND‐LVC were enrolled. Each participant underwent NIRF assessment via Holter monitoring and signal‐averaged ECG (SAECG) during two separate visits and a baseline cardiac MRI while receiving at least 6 months of proper medical therapy. Inclusion and exclusion criteria were rigorously applied to ensure the selection of stable, optimally managed patients. Statistical analysis was conducted with a power of 80% and significance level *α* = 0.05.

**Results:** Key findings included that right ventricular ejection fraction (RVEF) < 5% was a strong predictor of ventricular tachycardia (VT) (OR = 20.57, *p* = 0.014). Additionally, the presence of > premature ventricular contractions (PVCs)/24h was associated with a significantly higher risk of hospitalization for VT or VF (OR = 5.29, *p* = 0.028). QTc> ms was also a statistically significant predictor of arrhythmia‐related hospitalization (OR = 4.00). In contrast, left ventricular late gadolinium enhancement (LGE) and LVEF were not independently predictive in this cohort.

**Conclusions:** RVEF, PVC burden, and QTc interval emerged as significant non‐invasive predictors of arrhythmic events in patients with ND LVC. These findings underscore the importance of evaluation of NIRFs in this population to enable dynamic risk stratification and potentially guide the implementation of preventive strategies for SCD.

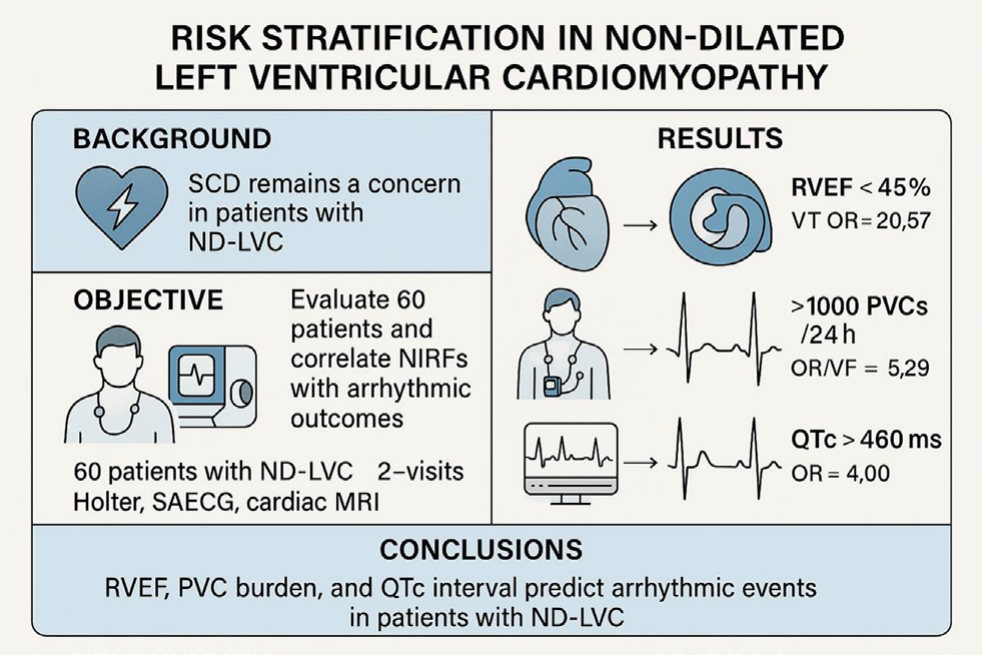

## 4658

### Preliminary Results of the ECHO‐LBBp Study: Impact of Echocardiographic LBBB Patterns on Immediate QRS Narrowing With LBB Pacing Versus CRT

#### Georgios Leventopoulos ^1^, Kassiani‐Maria Nastouli^1^, Maria Bozika^1^, Eleni Papastavrou^1^, Georgios Boliaris^1^, Kornilia Pepa^1^, Georgia Xygka^1^, Efthimia Kapsali^1^, Rafail Koros^1^, Angelos Perperis^1^, Ioanna Koniari^2^, Periklis Davlouros^1^

##### ^1^Department of Medicine, Division of Cardiology, University Hospital of Patras, Patras, Greece, ^2^Liverpool Centre for Cardiovascular Science, Liverpool L14 3PE, United Kingdom

**Background:** Preliminary results from the ECHO‐LBBp trial (NCT06689111) suggest that 2D strain echocardiography‐based mechanical characterization of left bundle branch block (LBBB) may better predict acute response to cardiac resynchronization therapy (CRT). Left bundle branch pacing (LBBp) has emerged as a physiological alternative to conventional biventricular pacing. This analysis compared immediate QRS duration changes following LBBp and CRT, stratified by typical or atypical mechanical LBBB patterns.

**Methods:** In this prospective, non‐randomized interventional study, 39 patients with heart failure and reduced ejection fraction (LVEF < 5%) undergoing CRT were analyzed. Patients were classified by echocardiography into typical or atypical mechanical LBBB patterns before implantation. Four groups were defined: LBBp recipients with typical LBBB pattern (*n* = 7), with atypical LBBB pattern (*n* = 9), CRT recipients with typical LBBB pattern (*n* = 12), and with atypical LBBB pattern (*n* = 8). Four patients who crossed over to LOT‐CRT were excluded from group comparisons. QRS duration was measured pre‐ and post‐implantation. Group comparisons of ΔQRS were conducted using ANOVA and *t* tests.

**Results:** QRS reduction differed significantly among groups (ANOVA *p* = 0.022). LBBp with typical pattern showed greater QRS narrowing than LBBp with atypical pattern (−51.4 ms vs. −33.3 ms, *p* = 0.037). No significant difference was observed between CRT subgroups (−29.5 ms vs. −25.0 ms, *p* = 0.598). Overall, LBBp patients had greater QRS reduction than CRT patients (−41.3 ms vs. −28.0 ms, *p* = 0.022) (Figure 1).

**Conclusion:** Echocardiographic LBBB pattern may predict acute electrical response. LBBp achieved superior QRS narrowing versus CRT, especially in patients with typical mechanical activation.

## 4674

### Exploring the Potential of Αrtificial Ιntelligence (AI) in Cardiology Education

#### Eleni Dafli ^1^, Panagiotis Bamidis^1^, Ioanna Dratsiou^1^

##### ^1^Lab of Medical Physics and Digital Innovation, School of Medicine, Aristotle University of Thessaloniki, Thessaloniki, Greece

**Introduction:** Artificial intelligence (AI) is rapidly becoming a cornerstone of innovation in medical and patient education. The aim of this study was to evaluate the ability of ChatGPT to simulate clinical reasoning and decision‐making by interacting with a wide range of Virtual Patient (VP) scenarios offered through the MobiVIP app. Another aspect of this study was the comparative assessment of the GPT‐3.5 and GPT‐4 performance in resolving VP cases.

**Materials and Methods:** The Mobile Virtual Patients Scenarios app (MobiViP) was developed by the Lab of Medical Physics and Digital Innovation at the School of Medicine, Aristotle University of Thessaloniki (AUTH), for educational purposes. An interactive, step‐by‐step process was followed in this study and each VP scenario node—a point of decision or question‐ was presented to ChatGPT in natural language. The ChatGPT versions 3.5 and 4.0 were utilized. The evaluation framework enabled a systematic assessment of ChatGPT's ability to simulate clinical reasoning and support decision‐making processes in VP scenarios.

**Results:** The freely available version of theChatGPT software (3.5), had a success rate of 83%. Among these correct answers, in a percentage of 16% the software faced technical issues, as the closed‐ended questions in the clinical problems were contradictory to the software's tendency to answer in a detailed manner. The improved version (4.0) diminished the errors to a percentage of just 5% (success rate of 95%) and faced technical issues in only 3% of the correct answers. ChatGPT answered correctly even the questions that included electrocardiograms and X‐rays.

**Conclusions:** AI‐powered tools like ChatGPT hold great promise for revolutionizing medical and patient education. ChatGPT 4.0 had an accuracy of 95% in resolving real‐life clinical scenarios. This study is important not only for the immediate results of the performance of Artificial Intelligence in resolving clinical problems, but also for the extensions it has in the possibility of strengthening modern medical education. The combination of using interactive applications such as Virtual Patients and Artificial Intelligence can support doctor and patient education in the context of the wider digital transformation of medical education.

## 4675

### Impact of Superior Vena Cava Isolation on Outcomes of Atrial Fibrillation Ablation: A Systematic Review and Meta‐Analysis

#### Michail Botis ^1^, Dimitrios Tsiachris^1^, Ioannis Doundoulakis^1^, Nikos Argyriou^1^, Aikaterini‐Eleftheria Karanikola^1^, Panagiotis Tsioufis^1^, Konstantinos Tsioufis^1^

##### ^1^First Department of Cardiology, Hippokration General Hospital, Athens, Greece

**Background:** Pulmonary vein (PV) isolation is the mainstay in atrial fibrillation (AF) ablation. However, additional arrhythmogenic foci seem to contribute to AF initiation and maintenance. A great proportion of those non‐PV foci have been reported to be located in the superior vena cava. We aimed to investigate the effectiveness of SVC isolation as an adjunctive therapy to PV isolation.

**Methods:** We performed a systematic review of MEDLINE and CENTRAL. Inclusion criteria were cohort studies with control group or randomized clinical trials, comparing patients undergoing PV isolation only and patients undergoing PV isolation with concurrent SVC isolation, in effects of freedom from atrial tachycardia.

**Results:** A total of 9 studies, incorporating 1.874 patients, were included. The combination of SVC and PV isolation in patients undergoing AF ablation was more effective than PV isolation alone (odds ratio = 0.72; 95% confidence interval, 0.54‐0.97). In a subgroup analysis, radiofrequency ablation demonstrated effectiveness (odds ratio = 0.71; 95% confidence interval, 0.52–0.96). Conversely, the use of cryoablation did not alter clinical outcomes (odds ratio = 0.69; 95% confidence interval, 0.13–3.61).

**Conclusions:** The outcomes of AF ablation are favorable when additional SVC isolation is conducted, using radiofrequency energy, rather than cryo energy, in terms of atrial tachycardia recurrence.

## 4679

### Impact of Energy Source for Pulmonary Vein Isolation on Heart Rate Variability and Autonomic Modulation

#### Aikaterini‐Eleftheria Karanikola ^1^, Dimitrios Tsiachris^1^, Michail Botis^1^, Athanasios Kordalis^1^, Ageliki Laina^1^, Christos‐Konstantinos Antoniou^1^, Maria Kouremeti^1^, Panagiotis Xydis^1^, Nikos Argyriou^1^, Ioannis Kachrimanidis^1^, Konstantina Aggeli^1^, Konstantinos Gatzoulis^1^, Konstantinos Tsioufis^1^

##### ^1^First Department of Cardiology, Hippokration General Hospital of Athens, Greece

**Introduction:** Autonomic nervous system modulation has emerged as a potential therapeutic target in atrial fibrillation (AF) management. Ganglionated plexi located within the left atrium may be affected by the energy delivered around the pulmonary veins during catheter ablation, potentially influencing autonomic tone. Heart rate variability (HRV) provides a non‐invasive assessment of autonomic function and has been previously utilized to evaluate autonomic changes post‐ablation. This study aimed to investigate differences in HRV parameters following pulmonary vein isolation (PVI) performed with either cryoballoon ablation (CBA) or pulsed field ablation (PFA).

**Methods:** In this prospective single‐center study, we included 17 patients treated with CBA (35.3% female, mean age: 63.2 ± 11.7 years, 82.4% paroxysmal AF, mean AF duration: 99.4 ± 84.4 months) and 10 patients treated with PFA (40% female, mean age: 65.5 ± 7.3 years, mean AF duration: 46.4 ± 44.8 months). Standard 24‐h Holter monitoring was performed before catheter ablation and repeated 3 months post‐procedure. Heart rate (HR) and HRV time‐domain parameters were analyzed and compared within and between the two groups.

**Results:** At 3‐month follow‐up, the CBA group (*N* = 17) showed significant HRV reductions, with decreased SDNN (Δ SDNN = −49.6 ms, *p* = 0.007) and SDANN (Δ SDANN = −40.4 ms, *p* = 0.009), and increased night HR (*p* = 0.002). A trend toward reduced SDNN index was noted (*p* = 0.067). Supraventricular extrasystoles burden and RMSSD/pNN50 showed no significant changes. Conversely, the PFA group (*N* = 10) showed no significant changes in HRV time‐domain indices or arrhythmia burden. CBA was associated with significantly greater reductions in SDNN (*p* = 0.006) and SDANN (*p* = 0.012) compared to PFA. No significant between‐group differences occurred for mean heart rate, RR interval, pNN50, RMSSD, or SDNN index.

**Conclusion:** These findings suggest that CBA may have a more pronounced effect on autonomic modulation compared to PFA in the early post‐ablation period. Prognostic implications include a potential for personalizing AF treatment based on the energy source used for PVI, particularly in patients where autonomic neuromodulation is a consideration. Future research should explore the integration of thermal and non‐thermal energy sources to optimize clinical outcomes.

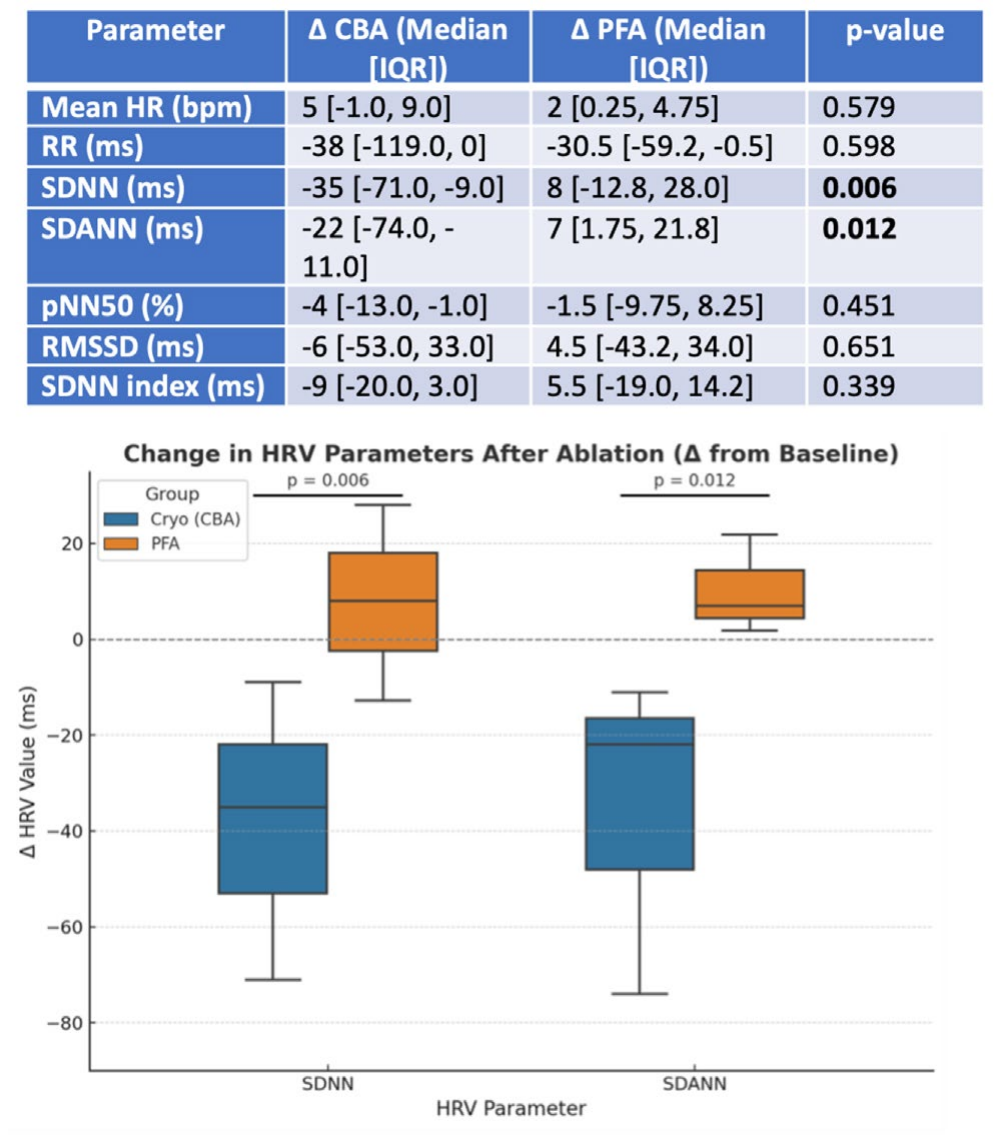

## 4680

### From Smartphone to STEMI: Enhancing ECG Interpretation Accuracy Using ESC Guidelines and AI Integration

#### Ioannis Kachrimanidis ^1^, Anastasios Apostolos^1^, Panagiotis K Vlachakis^1^, Panagiotis Tsioufis^1^, Nikolaos Argyriou^1^, Ioannis Skalidis^1^, Dimitrios Tsiachris^1^, Konstantinos Tsioufis^1^, Konstantinos Toutouzas^1^

##### ^1^Ippokrateio General Hospital of Athens, Athens, Greece

**Introduction:** Rapid and accurate diagnosis of ST‐Elevation Myocardial Infarction (STEMI) is essential to ensure timely reperfusion therapy and reduce adverse clinical outcomes. With the increasing availability of ECGs in digital and photographic formats, artificial intelligence (AI) offers a promising solution for augmenting clinical decision‐making, particularly in settings where specialist interpretation may not be immediately available. This study aimed to evaluate the diagnostic performance of structured ECG interpretation using smartphone‐acquired images, applying the European Society of Cardiology (ESC) STEMI criteria. Additionally, the project highlights the foundational role of labeled datasets in enabling AI‐based systems to reliably replicate expert‐level ECG interpretation and improve over time with expanded data.

**Method & Methodology:** A dataset of 100 12‐lead ECGs was compiled from patients presenting to the Emergency Dpt with ACS, using smartphone photography. Initial ECG interpretations were made based on standard clinical pattern recognition, followed by a re‐evaluation using ESC STEMI guidelines, which define new ST‐segment elevation at the J‐point in at least two contiguous leads: ≥ 2.5 mm in men < 0 years, ≥ 2 mm in men ≥ 40 years, ≥ 1.5 mm in women in V2–V3, and ≥ 1 mm in other leads, excluding cases with left bundle branch block or left ventricular hypertrophy. Each ECG was reclassified based on ESC criteria into categories: STEMI, non‐STEMI/ischemia, old myocardial infarction, or normal. Accuracy metrics were derived by comparing initial clinical impressions with ESC‐standard reclassification. This process was designed to inform future training of AI‐based diagnostic models, which rely on consistently annotated data for effective learning.

**Results:** Out of 100 ECGs, 80 were correctly classified according to ESC criteria, while 20 required reclassification, yielding an overall diagnostic accuracy of 80%. Specificity for non‐STEMI and normal ECGs was 100%, and 96% consistency was achieved for confirmed STEMI cases. The final diagnosis distribution included 48 STEMI, 40 normal ECGs, eight old infarcts, and four ischemic non‐STEMIs. Culprit vessel localization (e.g., LAD, RCA) remained accurate in clinically significant cases.

**Conclusion:** This project reinforces the diagnostic value of standardized, image‐based ECG interpretation and underlines the importance of dataset expansion to improve the reliability and generalizability of future AI‐assisted diagnostic tools.

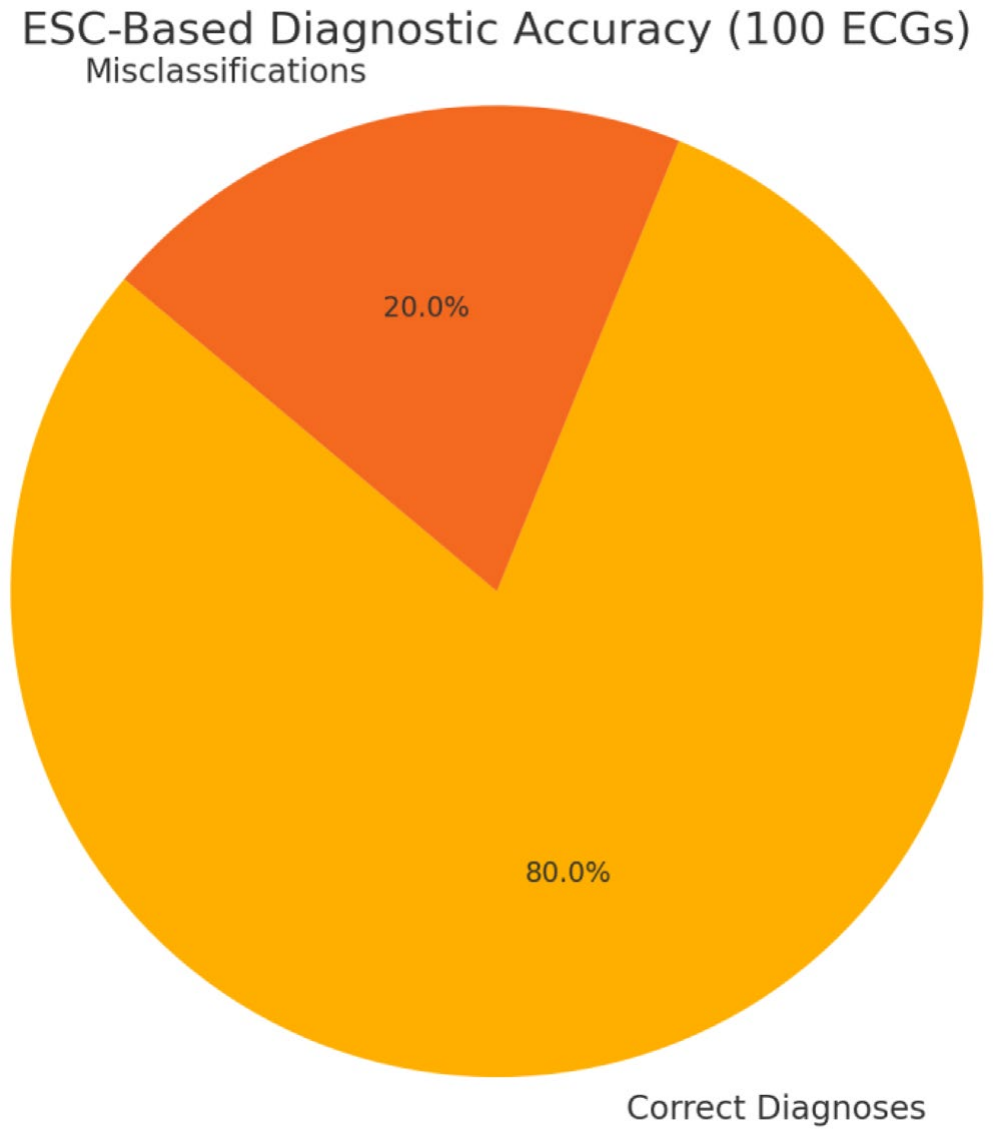

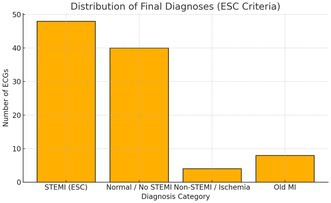

## 4583

### Cardiac Sympathetic Denervation in Patients With Refractory Ventricular Arrhythmias: A Single‐Center Experience

#### Ioannis Doundoulakis ^1^, Luigi Pannone^1^, Domenico Giovanni Della Rocca^1^, Giampaolo Vetta^1^, Gezim Bala^1^, Alexandre Almorad^1^, Erwin Ströker^1^, Juan Sieira^1^, Gian Battista Chierchia^1^, Andrea Sarkozy^1^, Mark La Meir^1^, Carlo De Asmundis^1^

##### ^1^Heart Rhythm Management Centre, Postgraduate Program in Cardiac Electrophysiology and Pacing, University Hospital Brussels‐Free University Brussels, European Reference Networks Guard‐Heart, Brussels, Belgium

**Introduction:** Cardiac sympathetic denervation (CSD) is a therapeutic option in patients with refractory ventricular arrhythmias (VAs). However, prospective data on long‐term outcomes in different population cohorts undergoing CSD are scarce. The aim of this study is to evaluate acute results, complications, and long‐term outcomes of CSD as a bailout therapy for VAs refractory to catheter ablation.

**Methods:** Adult patients who underwent CSD from October 2015 to February 2020 were retrospectively analyzed. Follow‐up was conducted via implantable cardioverter defibrillator (ICD) interrogation and reviewing medical records.

**Results:** A total of 15 patients (mean age 41.8 ± 21.1, 33.3% female) with nonischemic dilated cardiomyopathy (*N* = 5), idiopathic ventricular fibrillation (*N* = 4), catecholaminergic polymorphic ventricular tachycardia (*N* = 3), Long QT syndrome (*N* = 2) and Brugada syndrome overlap with long QT (*N* = 1) underwent CSD (left sided in 2 and bilateral in 13 patients). All patients had refractory VAs despite multiple anti‐arrhythmic drugs and prior VAs ablation (mean number of procedures was 3.4 ± 1.8). At a mean follow‐up of 80.3 ± 17.7 months, 66.7% and 80% of patients were free from any VAs or ICD shocks, respectively. Two patients experienced single ICD shock due to a monomorphic ventricular tachycardia (VT). One patient experienced multiple anti‐tachycardia pacing bursts and shocks due to polymorphic VT. No major complications of CSD occurred. No patient suffered from Horner syndrome.

**Conclusion:** The current retrospective analysis re‐emphasizes the role of surgical CSD to suppress VAs, when performed as a bailout therapy after previously unsuccessful catheter ablation. Further studies are needed to validate this finding in a prospective setting.

## 4591

### Corrected QT Interval as a Prognostic Factor in Patients with COVID‐19

#### Anna Kawińska ^1^, Patryk Siedlecki^1^, Ewa Mrozowska‐Peruga^1^, Piotr Lipiec^1^, Jarosław D. Kasprzak^2^, Małgorzata Kurpesa^1^

##### ^1^I Department and Chair of Cardiology, Bieganski Hospital, Medical University of Lodz, Poland, ^2^1st Department of Cardiology, Bieganski Hospital, Medical University in Lodz, Lodz, Poland

**Introduction:** A number of studies reported various ECG changes in COVID‐19 patients, but the problem still has not been fully described. The aim of the study was to investigate the prognostic value of the corrected QT interval (QTc) in COVID‐19 patients.

**Methods:** We investigated ECG changes in patients with COVID‐19 hospitalized in Cardiology Department from October 2020 to June 2021. Inclusion criteria: age ≥ 18 years, confirmed COVID‐19 disease. Exclusion criteria: inability to perform standard ECG, prior implantation of pacemaker or cardiac resynchronization therapy. On admission, standard 12‐lead ECG was performed using the tele‐ECG technique and sent to the telemedicine platform. ECG was analyzed by physicians blinded to clinical characteristics and further outcomes. Demographic, clinical data and laboratory test results have been collected. Information about outcomes such as death at any cause during hospitalization and within 18 months of follow‐up have been obtained. Patients were not treated by chloroquine or hydroxychloroquine. Spearman and rank‐biserial correlation tests were used for correlation analysis. Logistic regression to evaluate the association between QTc interval and mortality in univariate and multivariable models was used.

**Results:** Total of 201 patients were included to analysis (133 [66.17%] men vs. 68 [33.83%] women, mean age: 67.22 ± 13.39 years). All‐cause mortality during hospitalization was 32.84% and within 18 months it was 47.76%. Mean QTc was 456.21 ± 56.31 ms. QTc positively correlated with NT‐proBNP (*r* = 0.357; *p* < 0.001), IL‐6 (*r* = 0.215; *p* = 0.002), CRP (*r* = 0.147; *p* = 0.039), procalcitonin (*r* = 0.246; *p* < 0.001) and mortality within 18 months (*r* = −0.238; *p* = 0.004). There was inverse correlation between QTc and LVEF (*r* = −0.293; *p* < 0.001). In univariate analysis, QTc was associated with mortality within 18 months (OR = 1.006; 95% CI: 1.001–1.011; *p* = 0.028), but after multivariable adjustment it was not independently associated (OR = 0.998; 95% CI: 0.991–1.006; *p* = 0.652).

**Conclusions:** In patients with COVID‐19, QTc interval correlates with NT‐proBNP, markers of inflammation, and LVEF, and is associated with increased mortality within 18 months.

## 4604

### Evaluation of QRSD × Tpeak‐Tend(c)/QRSd × QTm (c) for Ventricular Arrhythmia Risk Stratification in Heart Failure Patients with ICD/CRT‐D

#### Dimitrios Sfairopoulos ^1^, Konstantinos Zekios^1^, Christos Katsouras^1,2^, Panagiotis Korantzopoulos^1,2^

##### ^1^First Department of Cardiology, University Hospital of Ioannina, 45500, Ioannina, Greece, ^2^Faculty of Medicine, School of Health Sciences, University of Ioannina, 45110, Ioannina, Greece

**Background:** Risk stratification for ventricular arrhythmias (VAs) remains a major challenge in HFrEF patients.

**Aim:** This study aimed to evaluate a novel electrocardiographic index, QRSD × Tpeak‐Tend(c)/QRSd × QTm(c), in relation to VA events during the first year after ICD/CRT‐D implantation in patients with HFrEF.

**Materials & Methods:** We retrospectively analyzed HFrEF patients with ICD/CRT‐D implantation. QRS duration (QRSd), Tpeak‐Tend, and QT intervals were measured in lead V5. For QRSd ≥ 120 ms, Bogossian's formula was used to calculate the modified QT (QTm). Tpeak‐Tend and QTm were corrected using Fridericia's formula, yielding Tpeak‐Tend(c) and QTm(c). QRS dispersion (QRSD) and QT dispersion (QTD) were calculated, and QRSD × Tpeak‐Tend(c)/QRSd × QTm(c) was derived.

**Results:** The 139 patients were divided into two groups based on VA event occurrence during the 1‐year follow‐up. VA events occurred in 22 (15.8%) patients; 117 (84.2%) had no events. LASSO regression identified 13 predictors explaining 50.5% of model deviance (*λ* = 0.0214). Backward stepwise selection retained seven predictors. Firth's penalized logistic regression showed that QRSD × Tpeak‐Tend(c)/QRSd × QTm(c) (OR 3.85, *p* < 0.001), RBBB, IVCD, and serum sodium were significantly associated with VA events. QTD, LAFB, and serum potassium were not statistically significant but remained in the model. The model showed excellent discrimination (AUC = 0.95), good calibration (Hosmer‐Lemeshow *p* = 0.936), and strong predictive accuracy (Brier score = 0.060).

**Conclusions:** QRSD × Tpeak‐Tend(c)/QRSd × QTm(c) was independently associated with VA events and may enhance risk stratification in this high‐risk population.

## 4611

### Stochastic Time Series Analysis of Atrial Fibrillatory Electrograms in Paroxysmal Atrial Fibrillation

#### Efstratios Karamanolis ^1^, George Giannakakis^2^, Adam Adamopoulos^3^, Dionyssios Leftheriotis^1^

##### ^1^Attikon University Hospital, Department of Cardiology, Athens, Greece, ^2^Foundation for Research and Technology—Hellas, Institute of Computer Science, Heraklion, Greece, ^3^Democritus University of Thrace, Department of Medicine Medical Physics Laboratory, Alexandroupolis, Greece

**Background/Introduction:** Regional dominant frequency and instantaneous frequency modulation of short fractionated atrial electrograms have been associated with ablation targets and the prognosis of atrial fibrillation (AF). However, inconsistent and not fully comprehensible results have been reported.

**Purpose:** To construct a simple computational algorithm for the analysis of continuous fibrillatory atrial electrograms and evaluate it in clinical practice among patients with paroxysmal AF.

**Methods:** Before the first cryo‐balloon ablation, 3‐min continuous local electrogram recordings during pace‐induced AF were obtained by the circumferential catheter from the left atrial appendage (LAA) and each pulmonary vein: left superior (LSPV), left inferior (LIPV), right superior (RSPV) and right inferior (RIPV). Following time‐ and frequency‐domain analysis, non‐linear detrended fluctuation analysis (DFA α) with analysis for short‐term (DFA α1) and long‐term fluctuations (DFA α2) was performed. The signals were detrended by subtracting the time series polynomial fit and bandpass filtered in the MATLAB environment (cut‐off voltage 0.1 mV to avoid scar and artifacts).

**Results:** Fifteen patients were studied. Among different atrial areas, higher mean peak rate (pr) along with lower standard deviation of between peaks intervals (sdpp) and root square of peak‐to‐peak differences (rmppd) were observed in the LAA and left veins (Figure 1). In patients with longer AF history (> 2 years), lower α, α1 and α2 exponents of DFA were observed (Figure 2).

**Conclusions:** This algorithm depicted dissimilar impact on atrial electrical activity between different atrial areas. Patients with longer AF history had less self‐similarity in local LAA electrograms. This might indicate possible ablation targets and reveal patients with greater AF burden and worse arrhythmic prognosis.

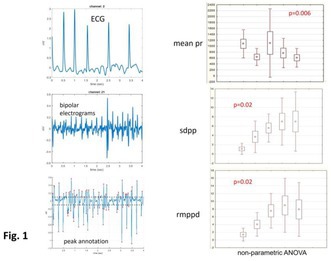

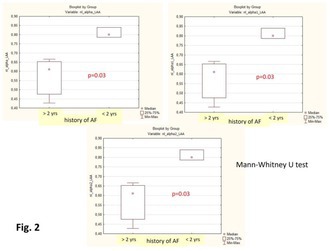

## 4617

### Do Slight Changes in PVC Stimulation Site Affect the Body Surface Potentials?

#### Lukáš Zelieska ^1^, Beáta Ondrušová^2^, Milan Tyšler^3^, Jorge Sanchéz^4^

##### ^1^1. Institute of Measurement Science, Slovak Academy of Sciences, Bratislava, Slovakia, 2. Faculty of Electrical Engineering and Information Technology, Slovak University of Technology, Bratislava, Slovakia, ^2^Johannes Kepler University, Linz, Austria, ^3^Institute of Measurement Science, Slovak Academy of Sciences, Bratislava, Slovakia, ^4^1. Centro de Investigación e Innovación en Bioingeniería, Universidad Politécnica de Valencia, Valencia, Spain, 2. Institute of Biomedical Engineering, Karlsruhe Institute of Technology, Karlsruhe, Germany

Computational modeling of cardiac electrophysiology provides insights into the propagation of electrical activity under normal and pathological conditions [1]. It is particularly useful for studying premature ventricular contractions (PVCs). Accurate positioning of the stimulation site is crucial, as even small inaccuracies can impact results. This study evaluates how closely located PVC sites affect body surface potential (BSP) patterns and compares them to a patient BSP.

In this study, we simulated PVCs originating from the true ablation site and four nearby sites (Figure, panel A) in the right ventricular septum, using a patient‐specific heart‐torso model derived from a CT scan. Ventricular coordinates defined tissue properties, and the pseudo‐bidomain model in openCARP simulated activation spread [2]. We compared simulated and measured BSPs from 128 torso electrodes recorded before RFA to identify the PVC origin that best matches the patient data. Our results (Figure, panel B, C) show the highest correlation (0.79) in BSPs when the PVC origin was site 3, while the lowest (0.15) was observed from site 4. BSPs from the PVCs at the ablation site had a slightly lower correlation (0.76) compared to site 3. These results suggest that even small shifts in the stimulation site can significantly influence BSP patterns. Further studies are required to evaluate the impact of such shifts on simulation results, incorporating additional sites and a larger patient cohort. Despite this, our findings suggest a pseudo‐bidomain model can accurately reproduce BSP from patient measurements.

[1] Trayanova, N.A., Lyon, A., Shade, J., Heijman, J. (2024). Computational Modeling of Cardiac Electrophysiology and Arrhythmogenesis: Toward Clinical Translation. In *Physiological Reviews*, 104 (3), 1265–1333. https://doi.org/10.1152/physrev.00017.2023.

[2] Plank, G., Loewe, A., Neic, A. et al. (2021). The openCARP Simulation Environment for Cardiac Electrophysiology. *Computer Methods and Programs in Biomedicine*, 208, p. 106223. https://doi.org/10.1016/j.cmpb.2021.106223.

